# Integrated metabolomic analysis and cytokine profiling define clusters of immuno-metabolic correlation in new-onset psoriasis

**DOI:** 10.1038/s41598-021-89925-7

**Published:** 2021-05-18

**Authors:** Elisabetta Tarentini, Giulia Odorici, Valeria Righi, Alessia Paganelli, Luca Giacomelli, Valentina Mirisola, Adele Mucci, Luisa Benassi, Elisabetta D’Aversa, Claudia Lasagni, Shaniko Kaleci, Eva Reali, Cristina Magnoni

**Affiliations:** 1 Dermatology Unit, Surgical, Medical and Dental Department of Morphological Sciences Related to Transplant, Oncology and Regenerative Medicine, University of Modena and Reggio Emilia, Modena, Italy; 2 Department for the Quality of Life Studies, University of Bologna, Rimini, Italy; 3 Polistudium SRL, Milan, Italy; 4 Department of Chemical and Geological Sciences, University of Modena and Reggio Emilia, Modena, Italy; 5 Department of Life Sciences and Biotechnology, University of Ferrara, Ferrara, Italy; 6 Department of Biotechnology and Biosciences, University of Milano-Bicocca, Milan, Italy

**Keywords:** Immunology, Skin diseases

## Abstract

The association between the metabolic profile and inflammatory cytokines in psoriasis is poorly understood. We analyzed the metabolic and cytokine/chemokine profiles in serum and skin from patients with new-onset psoriasis and healthy subjects (n = 7/group) by HR-MAS NMR and Bio-Plex immunoassay. Immuno-metabolic correlation matrix was analyzed in skin and serum to identify a potential immune-metabolic signature. Metabolomics analysis showed a significant increase in ascorbate and a decrease in scyllo-inositol, and a trend towards an increase in eight other metabolites in psoriatic skin. In serum, there was a significant increase of dimethylglycine and isoleucine. In parallel, psoriatic skin exhibited an increase of early inflammatory cytokines (IL-6, IL-8, TNF-α, IL-1β) and correlation analysis highlighted some major clusters of immune-metabolic correlations. A cluster comprising scyllo-inositol and lysine showed correlations with T-cell cytokines; a cluster comprising serine and taurine showed a negative correlation with early inflammatory cytokines (IL-6, G-CSF, CCL3). A strong positive correlation was enlightened between glutathione and inflammatory cytokines/angiogenesis promoters of psoriasis. The integration of metabolic and immune data indicated a molecular signature constituted by IL-6, IL1-ra, DMG, CCL4, Ile, Gly and IL-8, which could discriminate patients and healthy subjects and could represent a candidate tool in the diagnosis of new-onset psoriasis.

## Introduction

Psoriasis results from the interplay between genetic predisposing factors and environmental triggers, which lead to a self-sustained skin inflammatory loop in which autoimmune and autoinflammatory components co-exist^1,2^. Clinical and histological features of psoriatic skin lesions reflect some key mechanisms of the disease, such as hyper-proliferation, and angio-neogenesis. From the immunological point of view, psoriasis is characterized by profound alterations involving the sustained activation of the TNF-α/IL-23/IL-17 axis and high expression of early inflammatory cytokines^3–9^ that also extends at systemic levels.

Psoriatic inflammation has been proposed as a link between psoriasis and metabolic and cardiovascular comorbidities, and therefore the study of skin and serum metabolome in psoriatic patients and their correlation with inflammation is of the highest interest^10^.

Metabolomics define the global metabolite profile in a biological system and captures the metabolic perturbations driving physiological and disease statuses^11^. Metabolic profiling has been largely applied to characterize psoriasis at the serum level^12–15^. However, only one study that employs high-resolution magic-angle spinning (HR-MAS) NMR spectroscopy on psoriatic skin samples is available so far^16^.

Since metabolites are the end products and the most downstream representation of cellular processes, the metabolic profile can undergo variations according to the proliferative activity of T-cells, neutrophils and keratinocytes. In addition, in mouse models of psoriasis-like inflammation, it has been reported very recently that metabolic variation can also actively contribute to elicit pathogenic events that lead to sustained skin inflammation and autoimmunity^17^. For these reasons, there is an increasing need to shed light on the interplay between metabolites and inflammatory molecules in both psoriatic skin and in the systemic circulation.

We analyzed the metabolic and the cytokine profiles in serum and skin biopsies from patients with new-onset psoriasis (within the last 6 months) and from healthy subjects by using integrated approaches, namely NMR spectroscopy and Bioplex Pro Human Cytokine assay^18^, with the following objectives: (i) define the metabolic profile of skin and serum samples from patients with new-onset psoriasis; (ii) analyze the cytokine and chemokine profile of tissues and serum by multiple cytokine assay; and (iii) correlate the metabolic profile with the cytokine profile in psoriatic serum and skin samples. Finally, by integrating the metabolic and immune profiling, we sought to define an immuno-metabolic signature, which can help discriminate between new-onset patients and healthy subjects.

## Results

### Metabolomics characterization of new-onset psoriasis

#### Qualitative analysis defines the metabolic profile of psoriasis

From the HR-MAS NMR spectra, 34 metabolites were identified from skin samples, while in the serum NMR spectra, 36 metabolites were identified (Supplementary Table 1). The 1D ^1^H CPMG spectra are shown in Supplementary Figure 1.

#### Quantitative analysis detects metabolic variations in psoriasis patient skin and serum samples

An explorative study through unsupervised multivariate statistical analysis, namely principal component analysis (PCA) (Supplementary Figure 2a), was undertaken on the spectra of both skin and serum samples. While PCA did not show any substantial clustering among the sample of the two classes, the PLS-DA (Supplementary Figure 2b) scores plot showed two groups of clusters of skin samples. Scores move to positive values of the latent variables 1 (LV1) passing from healthy to psoriatic subjects (Q^2^ = 0.52) (Supplementary Figure 2B). Inspection of the loading profile of LV1 shows that Ala, Ac, PGA, Cho, Scy, Gly, Ser, Myo, Glc and His were higher in healthy tissues, whereas Asc and Tau are higher in psoriatic skin.

A less defined separation was observed in serum.

A quantitative analysis was performed, focusing on the metabolites highlighted by the supervised orthogonal projection to latent structure analysis (PLSDA) and metabolites that had at least one signal suitable for deconvolution.

**Figure 1:**
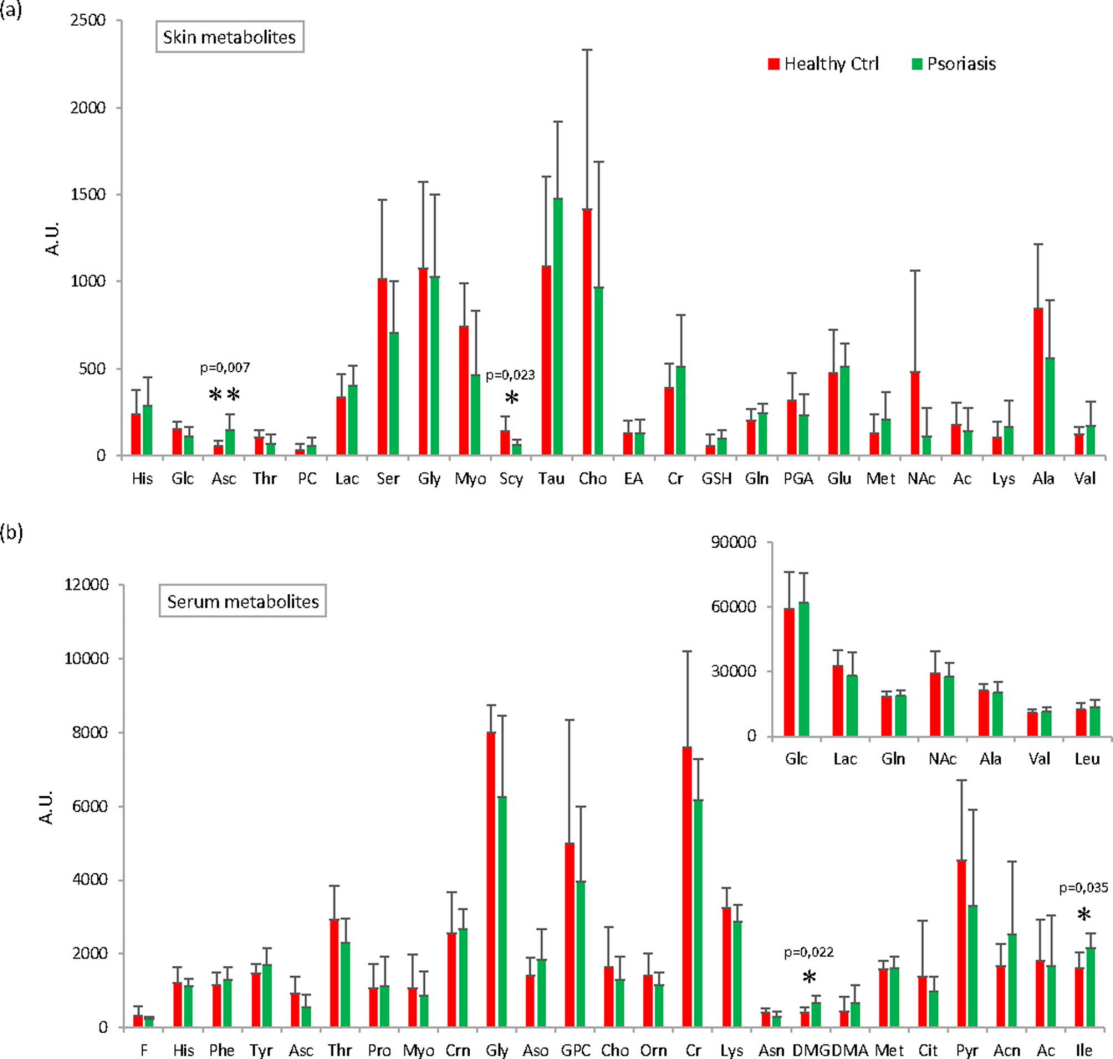
Relative quantification of metabolites after deconvolution of NMR signals. The bar plots show the relative amounts (mean ± standard errors) of metabolites identified in (**a**) skin and (**b**) in serum samples. (**b**) Some metabolites are reported in two bar charts with different y-axis expansions. Statistically significant (p < 0.05) changes in metabolite levels of Asc and Scy (**a**), DMG and Ile (**b**) detected by t-test are indicated by asterisks. Figure produced by Topspin (Bruker) Version 4.0.7 (https://www.bruker.com/content/bruker/int/en/products-and-solutions/magnetic-resonance/nmr-software.html). No permission required.

Deconvolution of spectral signals indicate that in psoriatic skin samples there was an increase in Asc, GSH, Lac, Tau, Cr, PC, Lys, Gln, Glu, Met and Val. A significant decrease in Scy (p = 0.023) and a decrease in Glc, Thr, PGA, Ser, Myo, Cho, NAc, Ac and Ala was also observed in skin psoriatic lesions (Fig. 1a).

Differences were then analyzed in serum samples showing a significant increase of dimethylglycine (DMG; p = 0.0228) and Ile (p = 0.0335) and trend towards an increase of Acn, Aso and DMA in psoriasis patients (Fig. 1b). A trend towards a decrease in psoriatic patient serum was observed for Asc, Thr, Gly, GPC, Cho, Asn, Cit and Pyr (Fig. 1b).

#### Chemometric analysis defines a trend of variation of the metabolic profile in psoriasis patients

**Figure 2:**
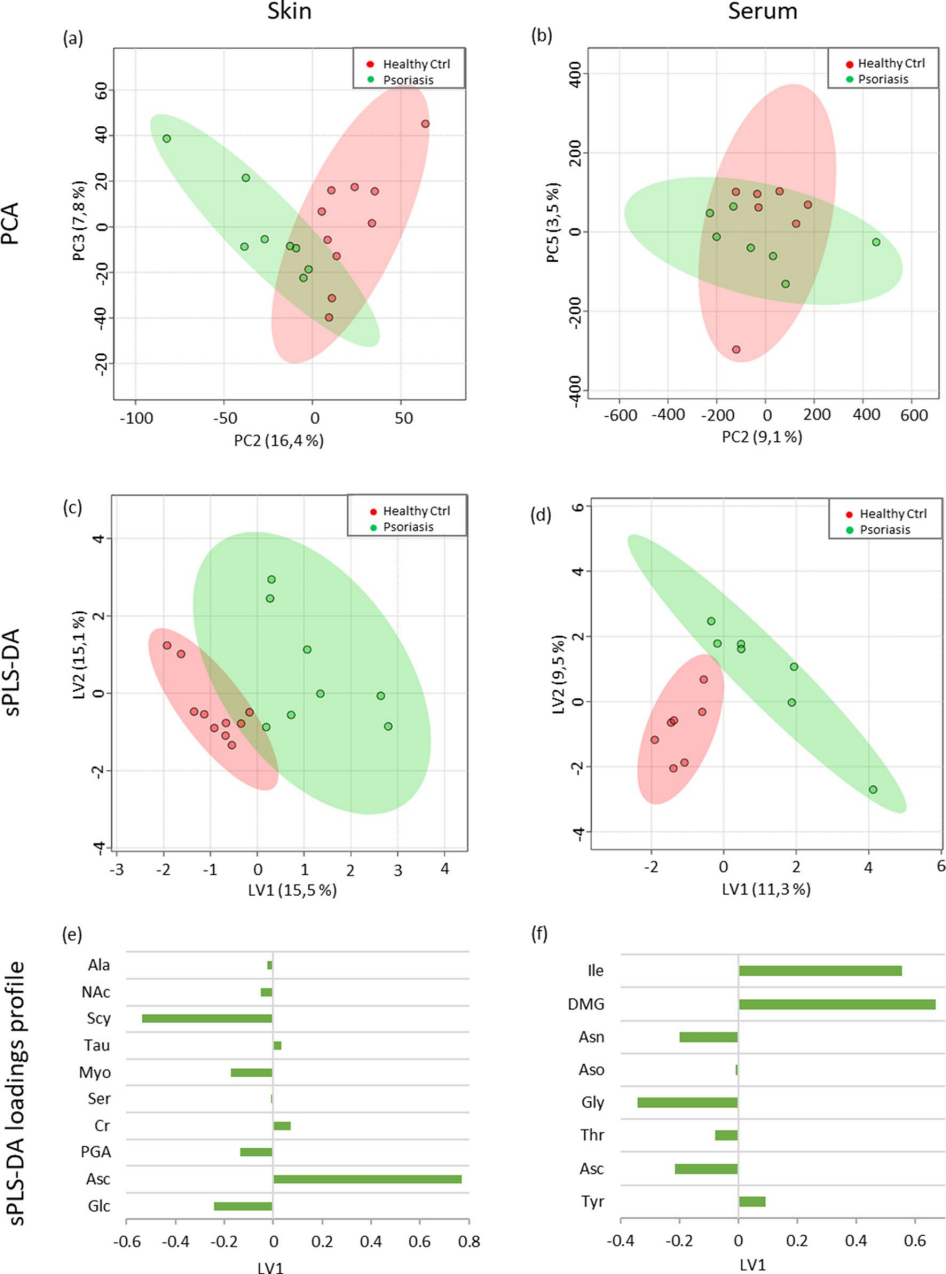
Scores plots of principal component analysis (PCA) of skin (**a**) and serum (**b**); and sparse partial least square-discriminant analysis (sPLS-DA) of skin (**c**) and serum (**d**) from deconvoluted NMR signals. Healthy control samples in red and psoriasis samples in green. sPLS-DA loading profiles of latent variable 1 (LV1) from skin (**e**) and serum samples (**f**). Figure produced by Microsoft Excel, Power Point, Microsoft Office Professional Plus 2019 (http://www.office.com). No permission required.

The unsupervised PCA analysis (Fig. 2a,b) of the quantitative metabolites data showed a trend of clustering for psoriatic and control samples. The sPLS-DA scores plots (Fig. 2c,d) showed a separation between the healthy and psoriatic groups, where psoriatic samples grouped together moving to positive values of LV1. The loading profiles of LV1 of sPLS-DA indicate Scy, Glc, Myo, PGA, NAc, Ala, Ser, Asc, Cr and Tau in the skin (Fig. 2e), and Ile, DMG, Tyr, Gly, Asn, Asc, Thr and Ser in the serum (Fig. 2f) as relevant metabolites for clustering patients with early psoriasis and healthy subjects.

### Cytokine profile in new-onset psoriasis

**Figure 3:**
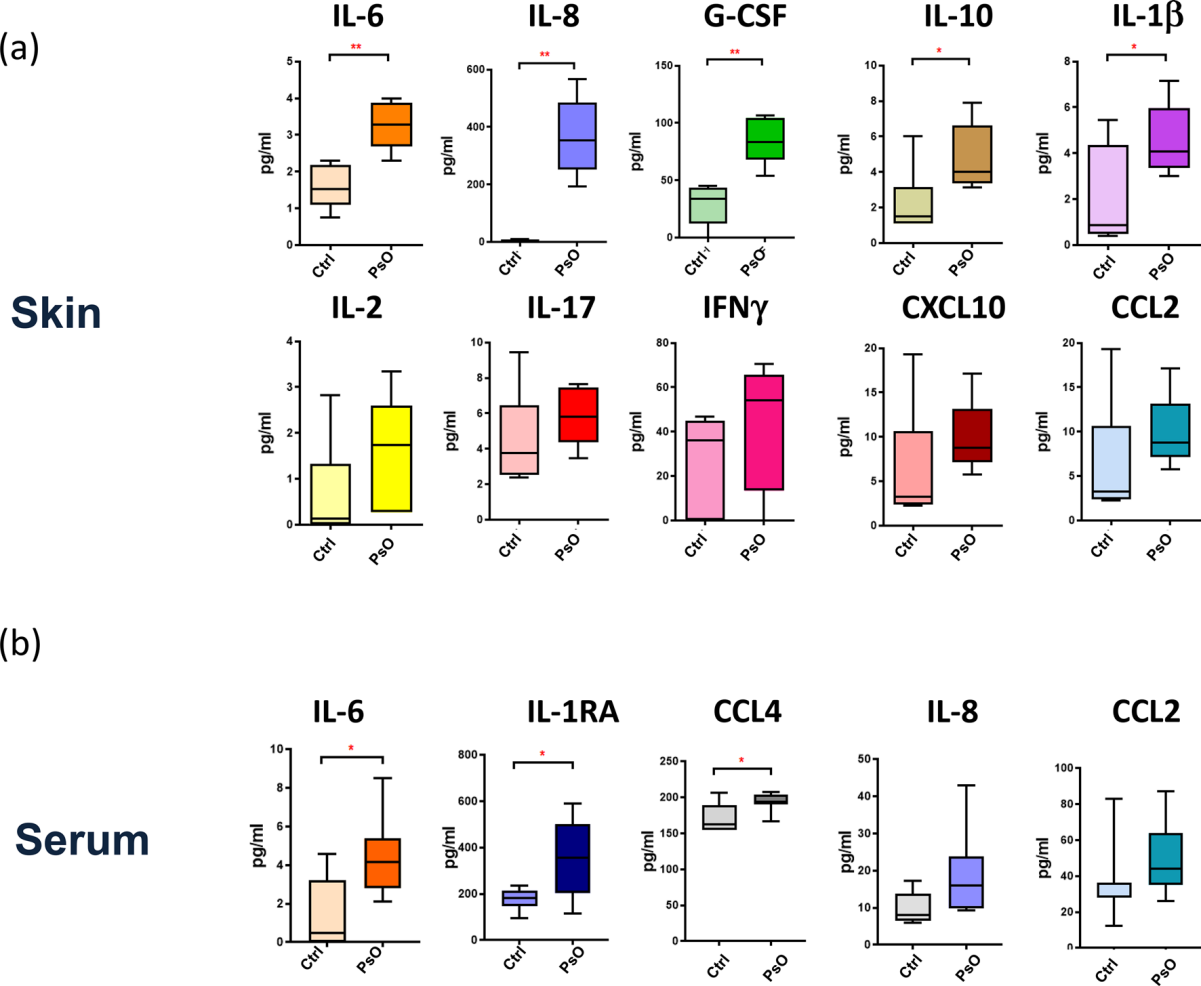
Cytokine and chemokine concentration (pg/ml) in skin (**a**) and serum (**b**) samples is represented as Box and Whisker Plot. Significance of the differences between psoriasis patients’ and healthy subjects’ control group was calculated using Student’s t-test for unpaired samples or Mann–Whitney test depending on the normality of data distribution. p < 0.05 was considered significant: *p < 0.05. **p < 0.01. Figure produced by Metaboanalyst Version 4.0 (https://www.metaboanalyst.ca) and Microsoft Excel, Office Professional Plus 2019 (http://www.office.com). No permission required.

We quantified 27 cytokines in skin and serum samples from psoriasis patients and the healthy subjects. In lesional skin from patients at early onset, we found a significant increase in the early phase inflammatory cytokines, namely IL-6 (p = 0.001), IL-8 (p = 0.0001), G-CSF (p = 0.001) TNF-α (p = 0.05) and a trend to increase for IL-1β (p = 0.05) and the anti-inflammatory cytokine IL-10 (p = 0.05) (Fig. 3a). Other cytokines, such as IL-17, IL-2, CXCL10, IFN-γ and CCL2, also showed a trend toward an increase. In serum, a significant increase of IL-1ra (p = 0.02), IL-6 (p = 0.01), as well as CCL4 (p = 0.03) was observed in psoriasis patients compared with healthy subjects (Fig. 3b).

### Correlation analysis identifies metabolite signatures associated with immune and inflammatory pathways in psoriasis patients

#### Correlation analysis of skin samples

**Figure 4:**
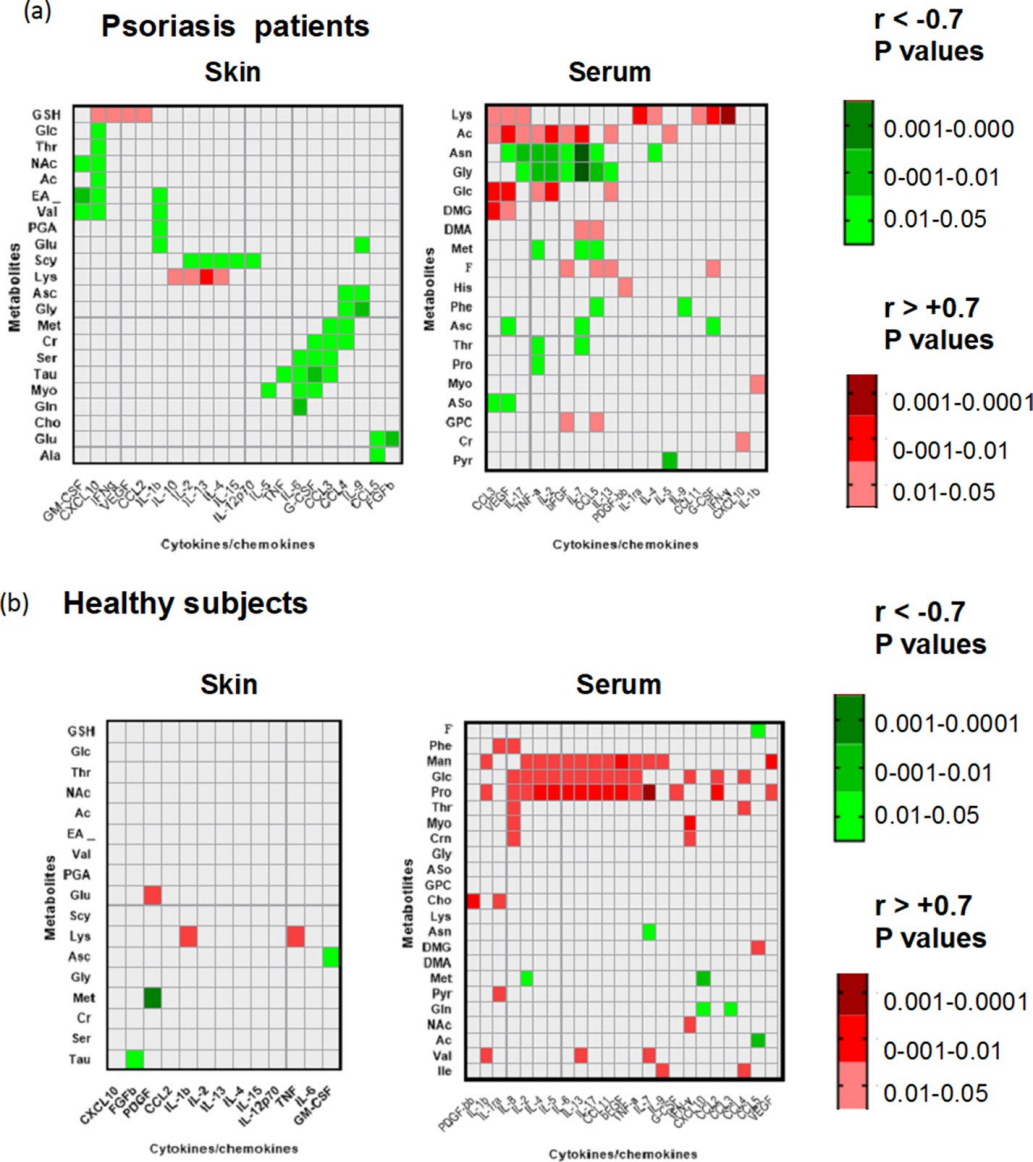
Heat maps of significant correlations between metabolites and cytokines/chemokines analyzed in skin biopsies and serum samples from psoriasis patients (**a**) or healthy control subjects (**b**). Pearson correlation coefficients (r) were used to express the correlation and r values <  − 0.7 and >  + 0.7 were selected to indicate negative and positive correlations, respectively. Green color gradients indicate negative correlations with values < 0.05 and red color gradient indicates positive correlations with p values < 0.05. r values not reaching significance correspond to uncolored squares. The complete table of r and p values of the correlations in the tissues analyzed is reported in supplemental data as supplementary table. Figure produced by Prism Software, version 8.3.0 Inc. USA (https://www.graphpad.com/). No permission required.

The analysis in the skin highlighted some major clusters of immuno-metabolic correlation (Fig. 4 and Supplementary Table 2). A first cluster shows that GSH positively correlates with the early inflammatory chemokines CCL2 and with VEGF, as well as with IFN-γ and CXCL10 of the Th1/Tc1/CXCL10 axis.

A second cluster of correlations included Scy and Lys, which correlated with IL-2, IL-13, IL-4.

Importantly, a third metabolites cluster comprises Ser and Tau showing a strong negative correlation with key inflammatory cytokines IL-6, G-CSF and CCL3 and the extended cluster comprising also Myo correlated with the cytokines IL-6 and G-CSF. Furthermore, it is important to mention smaller clusters of correlation comprising Met and Cr, which negatively correlated with CCL3, CCL4, Asc and Gly, which correlated negatively with IL-9 and CCL4.

Outside these clusters, we found a high number of correlations for CXCL10 showing a strong positive correlation with GSH but also a negative correlation with Glc, Thr, NAc, EA and Val. This high number of correlations with metabolites confirms the crucial role CXCL10 in skin pathology and evidences its relevance in the interconnections with the alterations of the skin metabolic profile and with glucose consumption.

Unlike psoriatic skin, samples from healthy subjects displayed a very restricted number of significant correlations between metabolites and cytokines/chemokines thus reinforcing the evidence of a strong connection between immune activation and metabolic changes in psoriatic skin.

#### Correlation analysis of serum samples

In serum samples from patients, we found a high number of significant correlations that differed from those observed in psoriatic skin samples.

Indeed, we identified a major cluster of correlation comprising Ac, Asn and Gly showing positive (Ac) and negative correlation (Asn and Gly) with key cytokine of the psoriasis pathology, such as IL-17, IFN-γ, IL-2, as well as with IL-7 and FGF. A sub-cluster comprising only Asn and Gly also correlated with CCL5 in addition to the above cytokine group. A second cluster included Lys, Ac, Glc and DMG showing positive correlation with CCL3 and VEGF. Glucose also showed a marked positive correlation with IL-2 and with TNF and IL-13. A smaller correlation cluster that includes DMA and Met with positive (DMA) and negative (met) correlation with IL-7 and CCL5 was also shown. IL-7 also showed negative correlation with Asc and Thr.

IL-7, therefore, emerges as a relevant cytokine at systemic level in psoriasis patients and a node that may link with metabolites pathways.

Similar to what observed in psoriatic skin lesion, serum Lys showed a significant positive correlation with IFN-γ, IL-1ra, GCSF, IL-17, IL-4, CCL3, CCL-11 and VEGF.

A very different pattern of correlation was observed in serum from healthy subject where it emerged one main cluster of correlation formed by Man, Glc and Pro that positively correlated with multiple inflammatory and T-cell cytokines. It is important to notice that also in this case IL-7 showed the highest level of correlation with metabolites Pro (p < 0.0005) and Man.

#### Identification of a potential immune-metabolic signature of new-onset psoriasis

**Figure 5:**
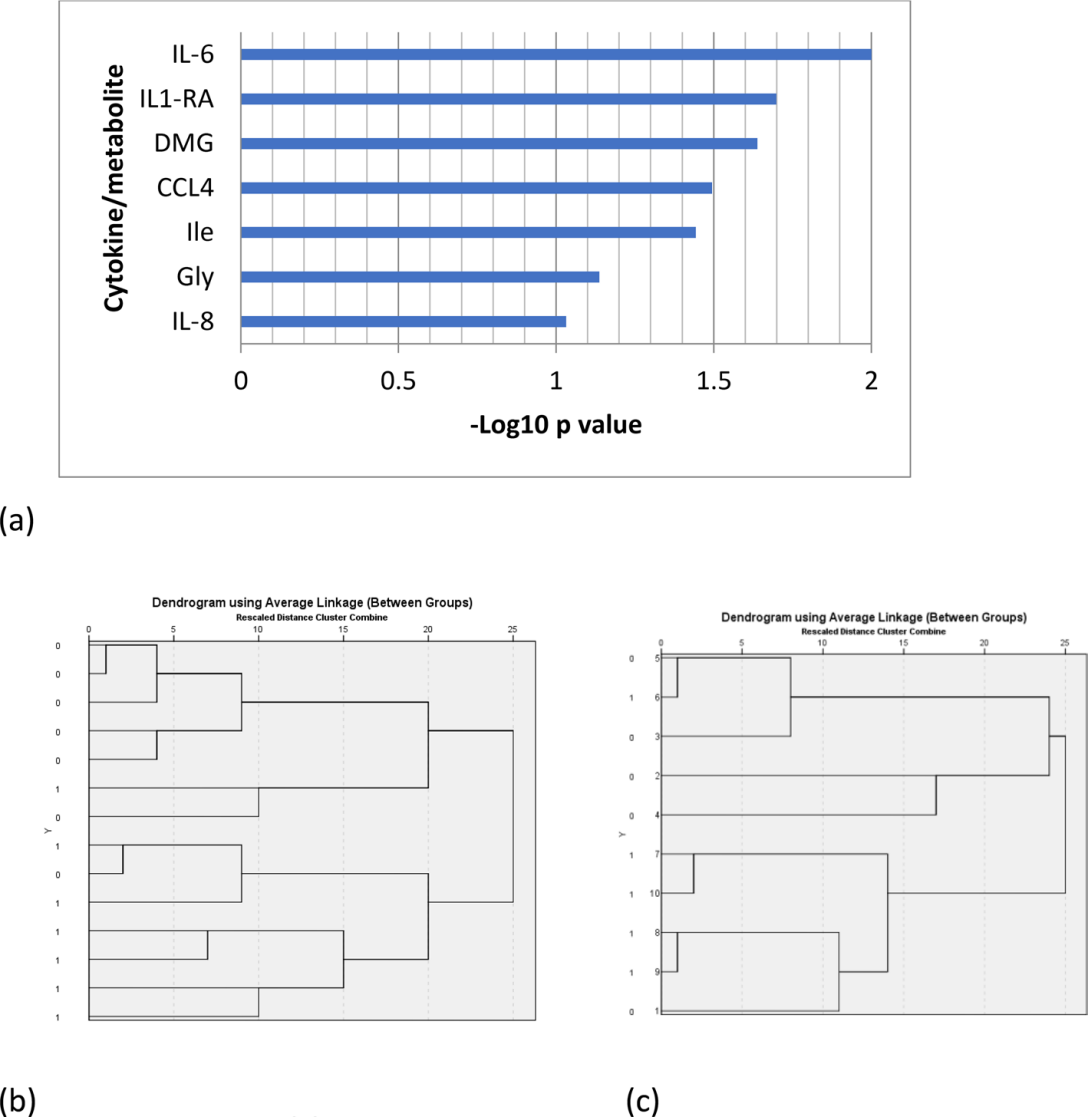
Candidate signature molecules were ranked for their potential patients/control discriminant capacity on the basis of the p-values of the difference between their concentration in patients and control groups (-Log10 p-values). (**a**). The discriminant power of the candidate signature was evaluated using hierarchical clustering in serum samples (**b**). Hierarchical clustering was applied also to tissue samples, using the 5 candidate molecules expressed also in tissue (IL-6, CCL4, IL1-ra, Gly and IL-8) (**c**). Figure produced by Metaboanalyst Version 4.0 (https://www.metaboanalyst.ca) and Microsoft Excel, Office Professional Plus 2019 (http://www.office.com). No permission required.

From the t-test between serum metabolites and cytokines from psoriasis patients and healthy volunteers, seven candidate signature molecules were obtained: DMG, Gly and Ile, together with IL-6, CCL4, IL1-ra, and IL-8. Molecules were ranked for their potential patients/control discriminant capacity on the basis of the p-values of the difference between their concentration in patients and control groups (Fig. 5a). By applying an unsupervised two-cluster analysis to serum samples using these signature molecules, two clusters were obtained, with fair cluster quality (average silhouette = 0.5) in which IL-6 emerged as the most important molecule. To evaluate the discriminant power of the signature, hierarchical clustering was used. Two main clusters were obtained, with correct assignment of 10/12 samples (83.3%) (Fig. 5b). Hierarchical clustering was also applied to skin samples, using the five signature molecules also expressed in the skin (IL-6, CCL4, IL1-ra, Gly and IL-8). Correct clustering of 8/10 samples (80.0%) was obtained (Fig. 5c), thus confirming the power of the immuno-metabolic signature to distinguish between patients with new-onset psoriasis and healthy subjects.

## Discussion

This study demonstrates a quantitative variation in the metabolic profile in both skin and serum samples of patients with new-onset psoriasis of moderate severity compared with healthy subjects. Metabolites that allowed the discrimination of psoriatic skin from healthy skin included Asc, Cr, Tau, Glc, PGA, Myo, Scy, NAc and Ala. Serum showed a different behavior and the metabolites that discriminate the two groups of samples were Asc, Thr, DMG, Gly, Asn and Ile. Data were confirmed by an unsupervised and supervised analysis. Some of these variations are consistent with some previous studies on either sera or lesional skin samples from psoriasis patients^19,20^. However, we did not take into account some potential confounding factors (e.g., dietary habits), and we cannot rule out that patients with more severe psoriasis might show a different metabolic profile.

In addition to this metabolomics data, here, we analyzed in parallel the variations of the metabolic and immune profile in both lesional skin and serum from patients^8^. These had the aim to start to explore the interplay between the well-characterized changes in the immune and inflammatory profile in psoriatic skin/sera and the emerging variations of the metabolic profile associated with the pathology.

Indeed, the alterations of the metabolic profile can be associated with the increased infiltration and activation of T-lymphocytes and inflammatory cells and with keratinocytes hyper-proliferation. The hyper-proliferative or hypermetabolic state in psoriatic skin can also explain the decrease of Myo and Scy, which are second messengers in the “phosphatidylinositol signaling system”, also involved in scavenging activity and membranes building^21^. In psoriatic tissue, the decrease of these molecules could reflect intensive cell signaling, as well as the massive need of antioxidants due to extensive tissue damage.

Similarly, a key role for Asc metabolism in psoriasis patients clearly emerges from our analysis in both psoriatic skin biopsies and serum. Asc is known to counteract the oxidative stress^22^ and represents an enzymatic cofactor of many biological processes. Moreover, in the skin, Asc may also influence morphology and differentiation of keratinocytes, as well as gene expression^23^. The increase of Asc in skin samples and the simultaneous decrease in sera shown in our study is in line with the results of a previous study and reinforces the evidence of a strong gradient of ascorbate from the blood to the epidermis as a possible compensatory mechanism to oxidative stress^24^.

Oxidative stress justifies the high concentration of Tau since this molecule has different biological roles, including the regulation of cell volume and anti-oxidative effects^25^.

Another key feature of psoriatic skin is a decrease of Glc level and a trend towards an increase of Lac. This is consistent with a high Glc consumption requested to sustain keratinocyte hyper-proliferation, and immune cell activation in psoriatic skin. The high levels of Lac in the skin most likely indicates production by metabolically active, proliferative cells, which undergo glycolysis even in the presence of oxygen. This phenomenon is known as ‘aerobic glycolysis’ or the ‘Warburg effect’^26–28^.

The analysis of an array of cytokines and chemokine expression showed higher concentrations of early inflammatory cytokines, in particular IL-6, which is significantly increased both in skin and blood serum and IL-8, TNF-α and IL-1 β which are increased in psoriatic skin. These cytokines are involved in the initial phase of psoriasis, characterized by early inflammatory events in keratinocytes and cells of the innate immunity before the amplification phase that expands Th17/Tc17 responses sustaining the chronic phase of inflammation^8,29^. The more pronounced increase in early inflammatory cytokines may be explained by the new onset of the disease, which could precede formation of established psoriatic plaque.

To establish the association between the relevant changes of the immune profile occurring in lesional skin (and serum) of psoriasis patients with the emerging changes in the metabolic profile the correlation analysis was performed enlightened some major clusters of correlation.

In psoriatic skin samples, the main cluster of correlations comprises Scy and Lys, which correlated with T-cell cytokines IL-2, IL-13 and IL-4.

Specifically, Scy negatively correlates with the cytokine levels, whereas Lys showed a marked positive correlation. The negative correlation with Scy and inflammatory T-cell cytokines can reflect the activation of phosphatidyl inositol-mediated signaling during intensive T-cell activation occurring in psoriatic skin^30^. Scy and Myo also showed negative correlation with other inflammatory cytokines.

On the other hand, Lys is essential for the crosslinking of collagen polypeptides, and its correlation with cytokines characteristic of T-cell and adaptive immunity likely reflects intense accumulation and activation of T-cells during the development of psoriatic lesions that could also be paralleled by tissue repair processes and collagen biosynthesis^29^. A second cluster focuses on GSH showing a marked positive correlation with the early inflammatory chemokine CCL2, and with VEGF as well as with IFN-γ and CXCL10 belonging to the CXCR3/CXCL10 axis with a major role in effector T-cell recruitment^31^. These cytokines are critical players in different phases of psoriasis and may associate with major alterations of the redox state of the tissue that could increase the requirement of an antioxidant, such as GSH. In parallel, VEGF could increase vascularization and oxygen supply^32,33^.

It is worth mentioning that, from this analysis, IL-7 emerges as nodal cytokine in the serum correlated with multiple metabolites in psoriasis patients and with proline in healthy subjects. A role for IL-7 in regulating immune cell metabolism has been previously mentioned only in few studies in the last decade and its role at the cross-road between T cells and systemic metabolic changes deserves further investigations.

The immuno-metabolic clusters emerging from this analysis indicate biochemical pathway associated with the initial phase of psoriasis development and may indicate the potential biomarkers of new-onset psoriasis diagnosis.

By integrating the results of metabolomics profiling and cytokine/chemokines profiling, we tried to define the possible immuno-metabolic signature that could characterize patients with new-onset psoriasis. In serum samples, a signature composed by IL-6, CCL4, Ile, IL1-ra, DMG, Gly and IL-8, was identified that could separate the samples into two distinct clusters by using unsupervised hierarchical clustering method. The same signature was confirmed in skin samples using the five putative signature molecules (IL-6, CCL4, IL-1ra, Gly and IL-8).

The molecule that primarily establishes a difference between patients with new-onset psoriasis and healthy subjects is IL-6. This molecule, in synergy with IL-1β, is considered a key driver to the differentiation of IL-17-secreting subset of T-helper cells^34^.IL-6 is increased in psoriatic skin and serum sample and is one of the possible mediators of systemic inflammation associated with psoriasis^8^. Indeed, according to the current model, inflammatory cytokines and chemokines produced at high level in the skin may also increase in the circulation thus contributing to systemic inflammation and creating a link with systemic metabolic effect^35^.

A very recent study in a mouse model of psoriasis-like inflammation suggests that IL-6 may be part of a loop that links metabolic alteration into the keratinocyte compartment with inflammatory cytokine production and the initiation events of the psoriatic plaque formation. such as the endocytosis of keratinocyte self-RNA by myeloid dendritic cells^17^. This may reinforce the interest about IL-6 in the early phase of psoriasis supporting the role as candidate diagnostic molecule in new-onset psoriasis.

In our cohort, the inflammatory cytokine CCL4 is another major signature molecule that discriminates between psoriasis patients and healthy subjects. It is worth to underline that, CCL4 polymorphisms and protein levels correlate with the psoriasis course and can also play a role in the development and/or progression of diabetes mellitus and atherosclerosis^36^^,^^37^. Therefore, it is possible to envisage that CCL4 may act as a possible node to link the presence of metabolic syndrome to psoriasis^38,39^. The IL-8 component of this signature is also well-known in psoriasis for its role in neutrophil recruitment and micro-abscesses formation^40^. Similarly, IL-1Ra is increased in inflammatory conditions to antagonize IL-1 activities.

From the metabolic profiling, it emerged that the level of Gly in skin and serum and the increase of DMG and Ile in the serum display a marked capacity of separating psoriasis and healthy control groups. The Gly decrease in skin and serum could reflect the higher cellular turnover. Indeed, Gly is consumed rapidly in proliferating keratinocytes and Gly depletion can limit the proliferation of rapidly-proliferating cells^41^. The amino acid catabolites DMG was significantly increased in serum of psoriasis patients. Beside of being part of the Gly, Ser and Thr metabolism, DMG is also part of the GSH metabolism and can derive from the re-methylation of homocysteine to methionine and is demethylated in the mitochondria, to Gly. Despite the link with GSH homeostasis with oxidative stress the association between increased DMG in serum and inflammation in human diseases still remains elusive^42,43^. Indeed, while the Ile level in human serum has already been reported as a possible biomarker in psoriasis^44^, a role for DMG in the diagnosis of psoriasis had not been reported previously.

**Figure 6:**
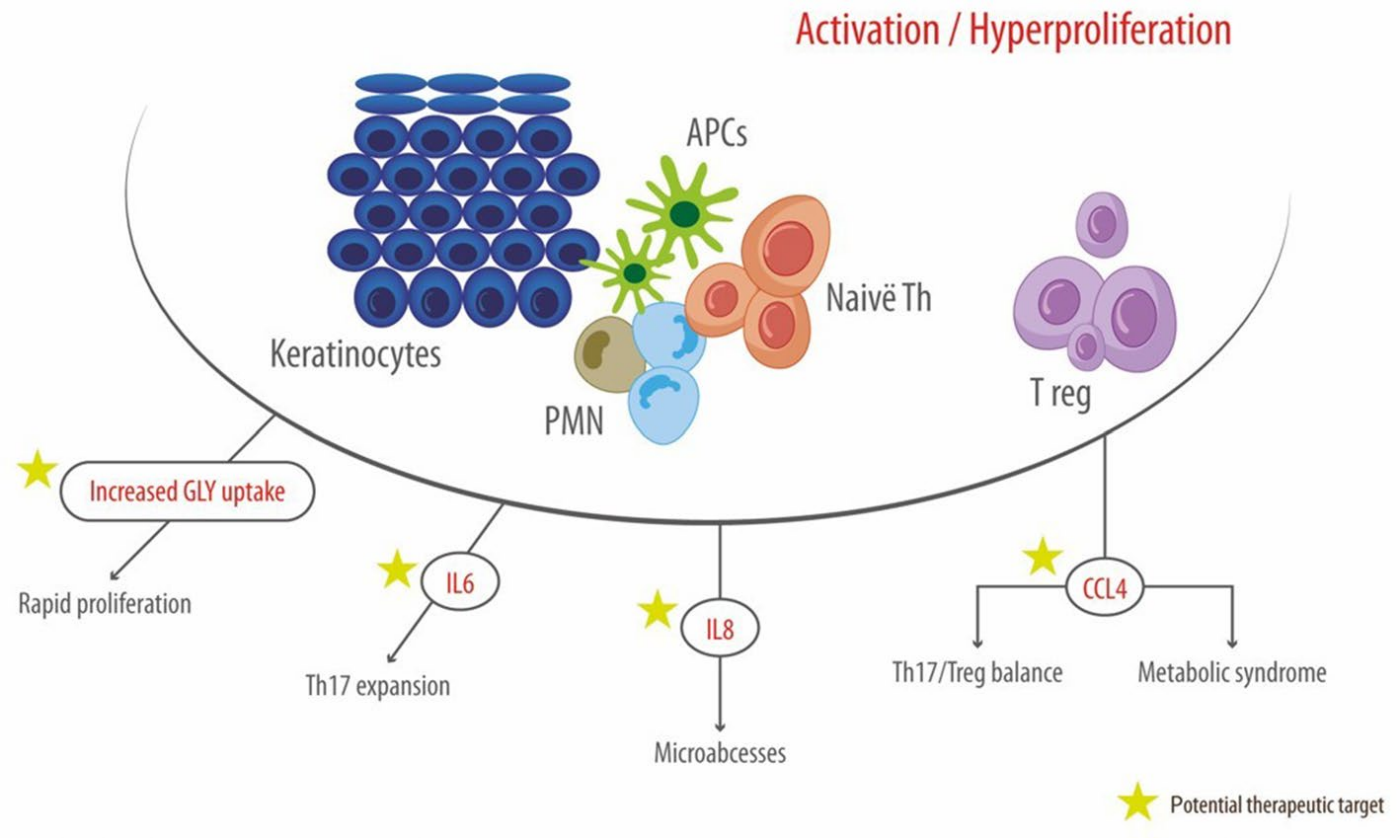
Candidate immune-metabolic signature of new-onset psoriasis. Figure produced by Adobe Illustrator 2020 (https://www.adobe.com/it/products/illustrator.html). No permission required.

In conclusion, our results provide a clear picture on the metabolic and inflammatory variation occurring in skin and serum of patients with new-onset psoriasis and define three clusters of correlation in the skin showing association between specific metabolites and either inflammatory or adaptive immune responses (Fig. 6). It finally allowed the identification of a potential immuno-metabolic signature of early psoriasis. Therefore, we consider that immuno-metabolic reprogramming may be worth further investigations for the comprehension of its therapeutic potential in new-onset psoriasis^45^.

## Patients and methods

### Study setting and population

The study was conducted at the Policlinico of Modena, University of Modena and Reggio Emilia (Modena, Italy) from 2016 to 2019.

The study was approved by the local Ethical Committee (pratica 8/16, Comitato Etico dell’Area Vasta Emilia Nord, Policlinico di Modena, Via Largo del Pozzo 71, 41124). The study was conducted according to the Helsinki Declaration. All patients signed an informed consent to the use of their data for research purposes.

In total, 18 consecutive subjects were enrolled from June 2016 to April 2017: 10 healthy subjects undergoing preemptive routine skin surgery for pigmented benign lesion removed for aesthetic reasons (e.g. dermal nevi), and eight patients who were diagnosed with psoriasis within the last 6 months before enrollment, by undergoing biopsy for histological confirmation. No patients had previously been treated for psoriasis. The medium PASI score was 8.4 ± 3.1. All participants provided skin samples, and 14 participants (seven psoriatic patients) also provided blood samples.

Baseline characteristics of the 10 healthy subjects were as follows: 6 males; mean age 59 ± 11 years; mean BMI 26 ± 3 kg/m^2^; two presented controlled hypertension and one dyslipidemia. On the other hand, baseline characteristics of the 8 patients were as follows: 5 males; mean age 55 ± 10 years; mean BMI 29 ± 6 kg/m^2^; one had a previous myocardial infarctio, one presented controlled hypertension, and one controlled hypertension + dyslipidemia.

### Procedures

A detailed description of the collection of skin biopsy (from normal skin adjacent to the pigmented benign lesion or from psoriatic lesion) and serum samples and NMR analysis is reported in the Supplementary Material^46,47^.

Metabolic profiles of skin and serum were obtained using a Bruker Avance III HD 600 MHz spectrometer^23,48,49^.

A total of 23 metabolites, selected according to low overlapping with the neighboring ones and to the results of the explorative analysis, were quantified^50^. Whole spectral profiles were analyzed, through principal component analysis (PCA) and sparse partial least squares discriminant analysis (sPLS-DA). MetaboAnalyst 4.0 was used for metabolomics data analysis^51^.

In the quantitative study, PCA and sPLS-DA was used to compare the areas obtained from deconvolution of the spectra of the two study classes.

The cytokine expression analysis was performed by the Bio-Plex Pro Human Cytokine 27-Plex Immunoassay (Bio-Rad Laboratories, Hercules, CA, USA) according to the manufacturer’s instructions. To investigate the correlation between metabolites levels and cytokines, Pearson correlation coefficients were calculated both in serum and in tissue samples from psoriasis patients and healthy volunteers (STATA14. Stata: Release 14. Statistical Software. College Station, TX: StataCorp LP). The heatmaps of data correlation between cytokines and metabolites were performed using MetaboAnalyst 4.0^51^.

Pearson’s correlation analysis was performed between metabolites and cytokine levels in the different tissues. R-values between 0.8 and 1 (for positive correlation) or between (− 0.8 and − 1) with values > 0.05 were considered the most significant correlations in skin and serum samples.

Clustering analysis was used to identify a possible immuno-metabolic signature.

A t-test between serum metabolites and cytokines from psoriasis patients and healthy volunteers was performed and all factors with ANOVA p < 0.100 were selected as candidate signature molecules. Molecules were ranked for − log10 p-value of the difference between their concentration in patients and control groups as a measure of their potential patients/control discriminating capacity. Unsupervised two-cluster analysis to skin samples using the candidate signature molecules emerging from serum sample analysis was performed. Hierarchical clustering to z-score standardized serum data, with between-groups linkage clustering method and Pearson correlation interval was performed to evaluate the discriminant power of the signature. The same method was used to clustering tissue samples, as validation of the signature. Clustering analyses were performed using IBM SPSS version 26.0.

## Supplementary Information

Supplementary Information 1.

Supplementary Information 2.

Supplementary Information 3.

Supplementary Information 4.

Supplementary Information 5.

## Abbreviations

F: Formate
His: Histidine
Phe: Phenyl-alanine
Tyr: Tyrosine
Man: Mannose
Glc: Glucose
GSH: Glutathione
Asc: Ascorbate
Thr: Threonine
Pro: Proline
PGA: Pyroglutamic acid (5-oxoproline)
PC: Phosphocholine
Lac: Lactate
Ser: Serine
Crn: Creatinine
Cr: Creatine
Myo: Myo-inositol
Gly: Glycine
Scy: Scyllo-inositol
Tau: Taurine
Aso: Anhydro sorbitol
GPC: Glycerol phosphorylcholine
Cho: Choline
Orn: Ornithine
EA: Ethanolamine
Lys: Lysine
Asn: Asparagine
DMG: Dimethylglycine
DMA: Dimethylamine
Cit: Citrate
Pyr: Pyruvate
Gln: Glutamine
Glu: Glutamate
Met: Methionine
Acn: Acetone
NAc: N-Acetyl
Ac: Acetate
Ala: Alanine
Val: Valine
Ile: Isoleucine
