# Supplementary material for: Integrated metabolomic analysis and cytokine profiling define clusters of immuno-metabolic correlation in new-onset psoriasis
Source: Sci Rep. 2021 May 18;11:10472. doi: 10.1038/s41598-021-89925-7 (PMC8131691; doi:10.1038/s41598-021-89925-7)
Supplement: Supplementary file 1 — Supplementary Information 1. [file 41598_2021_89925_MOESM1_ESM.docx]

Title page

Integrated analysis of metabolic and immune profile in new-onset psoriasis defines clusters of immune-metabolic correlation and indicates candidate diagnostic signature

Short title: Immuno-metabolic analysis and correlation in psoriasis patients

Elisabetta Tarentini^1^ (ORCHID ID: 0000-0001-6243-6166), Giulia Odorici^1^ (ORCHID ID: 0000-0002-5910-8994), Valeria Righi^2^ (ORCHID ID: 0000-0003-2394-8462), Alessia Paganelli^1^ (ORCHID ID: 0000-0002-0916-7769), Luca Giacomelli^3^ (ORCHID ID: 0000-0002-3600-5941), Valentina Mirisola^3^ (ORCHID ID: 0000-0003-0782-8225), Adele Mucci^4^ (ORCHID ID: 0000-0003-3303-8761), Luisa Benassi^1^ (ORCHID ID: 0000-0002-7866-2026), Elisabetta D’Aversa^5^ (ORCHID ID: 0000-0003-3058-5866), Claudia Lasagni^1^ (ORCHID ID: 0000-0002-0370-6602), Shaniko Kaleci^1^ (ORCHID ID: 0000-0002-1166-2961), Eva Reali^5†^ (ORCHID ID: 0000-0003-1900-1356) and Cristina Magnoni^1†*^ (ORCHID ID: 0000-0002-6081-4925)

1 Dermatology Unit; Surgical, Medical and Dental Department of Morphological Sciences related to Transplant, Oncology and Regenerative Medicine, University of Modena and Reggio Emilia, Modena, Italy

2 Department for the Quality of Life Studies, University of Bologna, Rimini, Italy

3 Polistudium SRL, Milan, Italy

4 Department of Chemical and Geological Sciences, University of Modena and Reggio Emilia, Modena, Italy

5 Department of Biotechnology and Biosciences, University of Milano-Bicocca, Milano, Italy

†These authors contributed equally to this manuscript.

* Correspondence: Cristina Magnoni, M.D. email: cristina.magnoni@unimore.it; Tel.: +39 0594222347

Received: date; Accepted: date; Published: date

Appendix A

Procedures

All skin samples were collected by punch biopsy of a 6-mm diameter and sectioned into two parts. One part underwent routine histological assessment while the other part was dissected to separate skin from adipose tissue, directly frozen in liquid nitrogen and stored at −80 °C until analysis. The serum samples were obtained from the same subjects, centrifuging the whole blood for 10 min at 1800 rpm after standing for 30 min at room temperature. The serum aliquots were stored at −80 °C. Before NMR acquisition, each serum sample was prepared adding 350 µl of serum aliquot to 350 µl of Verbr Buffer Plasma (Bruker BioSpin GmbH). After centrifugation at 12,000g × 5 min, 600 µl of supernatant was transferred into 5 mm NMR tube [19,20].

*NMR data acquisition*

Metabolic profiles of skin and serum were obtained using a Bruker Avance III HD 600 MHz spectrometer, operating at 600.13 and 150.90 MHz, for ^1^H and ^13^C, respectively, and equipped with a Bruker Cooling Unit for temperature control. Each skin sample was included in a 50 μl MAS zirconia rotor (4 mm OD) with 10 μl of deuterated water (D_2_O) and transferred into the cooled probe (278 K). All HR-MAS NMR measurements were performed at 278 K to prevent skin degradation [21], spinning the samples at 4000 Hz. The experiments and the experimental parameters were those previously described [22], apart from the number of scans of the water-suppressed spin-echo CPMG sequence (cpmgpr), which was 512.

NMR-based metabolic analyses were performed on serum samples at 310 K [19,23]. Three 1D ^1^H NMR experiments were carried out using (i) a composite pulse sequence (zgcppr), with 4 s water-pre-saturation during relaxation delay, 24 kHz spectral width, 32 k data points and 64 scans; (ii) a water-suppressed spin-echo CPMG sequence (cpmgpr) with 4 s water pre-saturation during relaxation delay, 1 ms echo time (τ), and 360 ms total spin–spin relaxation delay (2 nτ), 24 kHz spectral width, 32 k data points and 128 scans; (iii) a Nuclear Overhauser Enhancement SpectroscopY (NOESY)-presat sequence (noesypr1d) with 4 s relaxation delay, 0.01 s mixing time, 0.3 Hz line broadening, 24 kHz spectral width, 32 k data points and 64 scans.

2D experiments COSY, TOCSY and HSQC were performed to characterize the metabolome of skin and serum samples.

*NMR Data processing and statistical analysis*

Nearly 50 metabolites were identified on the basis of the chemical shifts, signal shapes, H,H and H,C correlations and comparison with spectral data reported in HMDB and BMRB data banks as previously reported [22]. ^1^H CPMG spectra were corrected for phase and baseline distortion, then normalized (with respect to skin weight for biopsies samples and to range area between 4.8 and 0.5 ppm for serum samples) and binned (δ 0.01 ppm) and used in the explorative analysis.

A total of 23 metabolites, selected on the basis of the low overlapping with the neighboring ones and of the results of the explorative analysis, were quantified in ^1^H CPMG spectra using Mnova 14 software (MestReNova, ver. 14. 0, 4-18998, 2018 Mestrelab Research S. L.; Santiago de Compostela, Spain). The relative concentrations of these metabolites were estimated using the areas of selected peaks, obtained by deconvolution. An automated/controlled fitting routine was employed, applying the Levenberg-Marquardt algorithm after manual peak selection, adjusting peak positions, intensities, line widths and Lorentzian/Gaussian ratios, until the residual spectrum was minimized [24].

Data were reported as mean ± standard error (arbitrary units [AU]). For Student’s t-test, paired two-sample test was used to determine the means. p<0.05 was considered statistically significant.

Whole spectral profiles were analyzed, after pareto scaling, through multivariate statistical analysis; in particular, principal component analysis (PCA) and sparse partial least squares discriminant analysis (sPLS-DA), excluding the regions with residual FA, squalene mepivacaine and exogenous alcohol signals. MetaboAnalyst 4.0, a comprehensive server, was used for metabolomic data analysis [25].

In the quantitative study, PCA and sPLS-DA was used to compare the areas obtained from deconvolution of the spectra of the two study classes, after pareto scaling, and using MetaboAnalyst 4.0 [25] also in this case.

*Cytokine expression and statistical analysis*

The cytokine expression analysis was performed by the Bio-Plex Pro Human Cytokine 27-Plex Immunoassay (Bio-Rad Laboratories, Hercules, CA, USA) according to the manufacturer’s instructions. The kit contains PDGF-β, IL-1β, IL-1ra, IL-2, IL-4, IL-5, IL-6, IL-7, IL-8, IL-9, IL-10, IL-12p70, IL- 13, IL-15, IL-17A, b-FGF, Eotaxin (CCL11), G-CSF, GM-CSF, IFN-γ, IP-10 (CXCL10), MCP-1 (CCL2), MIP-1α (CCL3), MIP-1β (CCL4), RANTES (CCL5), TNF-α and VEGF. Not all the samples allowed for a sufficient quantity of material and the analysis was performed on 11 skin samples and 14 serum samples in total.

Serum samples were diluted 1:3 v/v with the kit's sample diluent. Skin tissue lysates were prepared using the Bio-Plex cell lysis kit according to the manufacturer’s protocols and cytokines concentrations were measured. Tissue lysate at the appropriate protein concentration was added with an equal volume of Bio-Plex samples diluent and tested after storage at -20°C. Standard calibration curves were obtained using the standards diluted with 500 μL of the sample diluent and a series of eight dilutions. Each measurement was performed in triplicates. Quantification of cytokines content was achieved using the Bio-Plex 200 System, and the results were calculated using Bio-Plex Manager 6.0 software (Bio-Rad Laboratories). Cytokines levels were registered as 0 when were lower than the levels method sensitivity.

Baseline concentrations of cytokines were determined using internal standards according to manufacturer’s instructions. Concentration in the samples was calculated for each cytokine. The Shapiro-Wilk test was used to evaluate the Gaussian distribution of overall data. Statistical comparisons between the group of psoriatic patients and healthy controls were then calculated by using Student’s t-test depending on data’s Gaussian distribution and exact p-values were calculated.

*Correlation analysis between metabolites and cytokines*

To investigate the correlation between metabolites levels and cytokines, Pearson correlation coefficients were calculated both in serum and in tissue samples from psoriasis patients and healthy volunteers (STATA14. Stata: Release 14. Statistical Software. College Station, TX: StataCorp LP).

The heatmaps of data correlation between cytokines and metabolites were performed using MetaboAnalyst 4.0 [25].

r-values belonging to two-sided 5% of the extreme values of the Gaussian curve of the coefficients were considered the most significant correlations in skin and serum samples.

Clustering analysis was used to identify a possible predictive immune-metabolic signature.

A t-test between serum metabolites and cytokines from psoriasis patients and healthy volunteers was performed and all factors with ANOVA p<0.100 were selected as predictors. Unsupervised two-clusters analysis to serum samples using these predictors expression was performed. Hierarchical clustering to z-score standardized serum data, with between-groups linkage clustering method and Pearson correlation interval was performed to evaluate the discriminant power of the predictors. The same method was used to clustering tissue samples, as validation of the signature. Clustering analyses were performed using IBM SPSS version 26.0.
