# Supplementary material for: Integrated metabolomic analysis and cytokine profiling define clusters of immuno-metabolic correlation in new-onset psoriasis
Source: Sci Rep. 2021 May 18;11:10472. doi: 10.1038/s41598-021-89925-7 (PMC8131691; doi:10.1038/s41598-021-89925-7)

Figure S1. 1D ^1^H CPMG HR-MAS NMR spectra from (a) healthy controls and (b) psoriatic skin samples; liquid state NMR spectra from (c) healthy controls and (d) psoriatic serum samples. The most visible metabolites are labelled: [Glc] glucose; [Asc] ascorbate; [Thr] threonine; [Lac] lactate; [Crn] creatinine; [Ser] serine; [Gly] glycine; [Tau] taurine; [Scy] scyllo-inositol; [ChoCC] choline-containing compound; [Cr] creatine; [Glu] glutamate; [NAc] N-acetate; [Ac] acetate; [Ala] alanine; [Val] valine; and [Lip] Lipids.


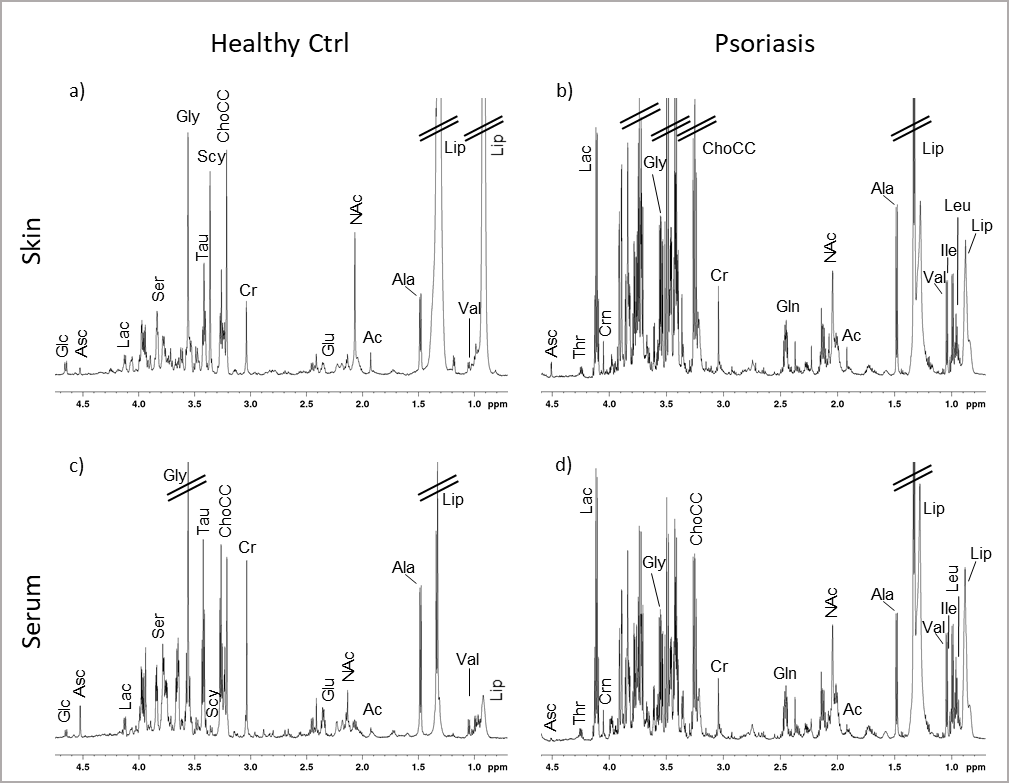

Supplement: Supplementary file 2 — Supplementary Information 2. [file 41598_2021_89925_MOESM2_ESM.docx]
