# Supplementary material for: Integrated metabolomic analysis and cytokine profiling define clusters of immuno-metabolic correlation in new-onset psoriasis
Source: Sci Rep. 2021 May 18;11:10472. doi: 10.1038/s41598-021-89925-7 (PMC8131691; doi:10.1038/s41598-021-89925-7)

Figure S2. Scores plots obtained by chemometrics analysis on skin. Principal component analysis (PCA) (A) and partial least square-discriminant analysis PLS-DA. (B) run on ^1^H CPMG NMR spectra. Healthy control samples in red and psoriasis samples in green.


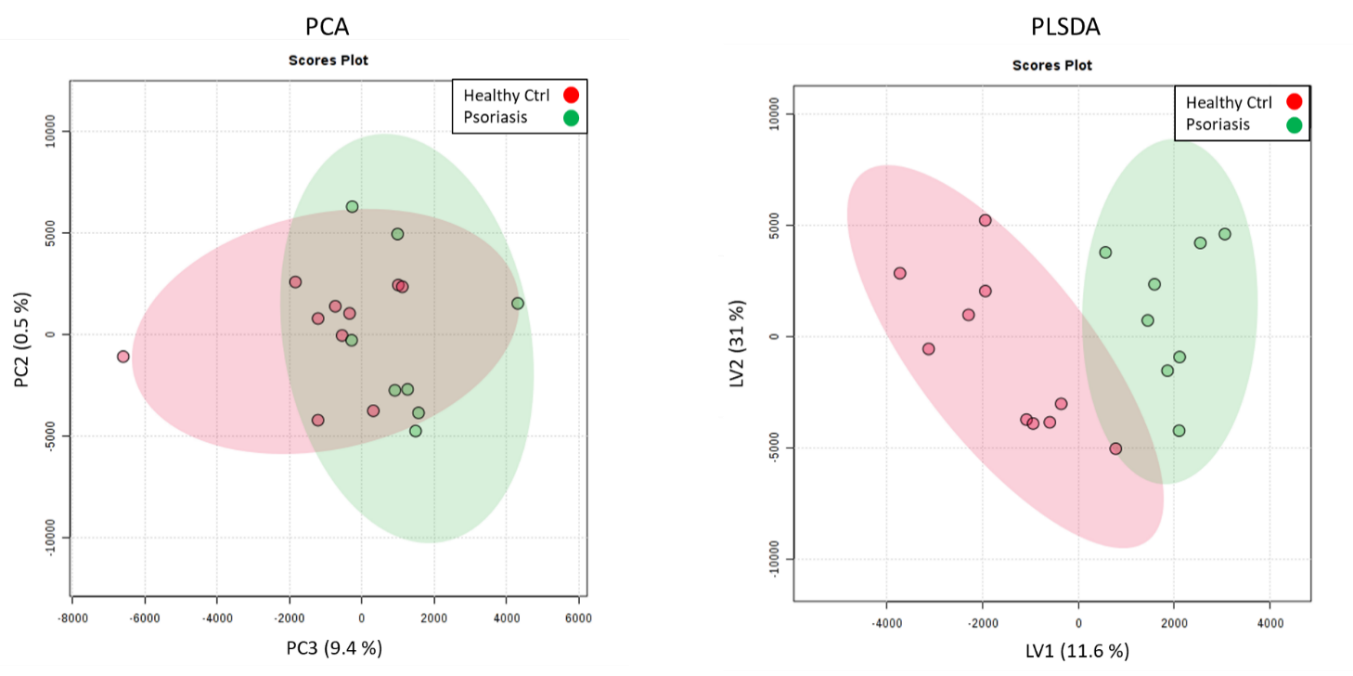

Supplement: Supplementary file 3 — Supplementary Information 3. [file 41598_2021_89925_MOESM3_ESM.docx]
