# Supplementary material for: Integrated metabolomic analysis and cytokine profiling define clusters of immuno-metabolic correlation in new-onset psoriasis
Source: Sci Rep. 2021 May 18;11:10472. doi: 10.1038/s41598-021-89925-7 (PMC8131691; doi:10.1038/s41598-021-89925-7)
Supplement: Supplementary file 4 — Supplementary Information 4. [file 41598_2021_89925_MOESM4_ESM.docx]

**Table A1. List of ^1^H and ^13^C chemical Shift (δ, ppm) of metabolites.**

|  | **Metabolites** | **Tissue (T) or**  **Serum (S)** | **δ^1^H**^a^ | **δ^13^C**^b^ | **Assignment** |
| --- | --- | --- | --- | --- | --- |
| **1** | Fatty acids | T and S | 0.89 | 16.7 | CH_3_ |
|  |  |  | 1.32–1.26 | 25.4, 32.6, 34.2 | (CH_2_)_n_ |
|  |  |  | 1.57 | 27.5 | CH_2_-CH_2_-C=O |
|  |  |  | 2.02 | 29.7 | CH_2_CH=CH |
|  |  |  | 2.25 | 36.2 | CH_2_-C=O |
|  |  |  | 2.76 | 28.0 | =CH-CH_2_-CH= |
|  |  |  | 5.30 | 130.7, 132.3 | -CH=CH- |
| **2** | Isoleucine | T and S | 0.94 (t) | 13.8 | δ-CH_3_ |
|  |  |  | 1.01 (d) | 17.2 | γ−CH_3_ |
|  |  |  | 1.45 |  | γ-CH_2_ |
|  |  |  | 1.97 |  | β−CH |
|  |  |  | 3.67 |  | α−CH |
| **3** | Leucine | T and S | 0.96 | 23.6 | δ-CH_3_ |
|  |  |  | 0.97 | 24.8 | δ−CH_3_ |
|  |  |  | 1.71 | 42.8 | β−CH_2_ |
|  |  |  | 1.73 |  | γ−CH |
|  |  |  | 3.72 | c | α−CH |
| **4** | Valine | T and S | 0.98 (d) | 19,3 | γ−CH_3_ |
|  |  |  | 1.04 (d) | 20.7 | γ−CH_3_ |
|  |  |  | 2.28 |  | β−CH |
|  |  |  | 3.61 | c | α-CH |
| **5** | Lactate | T and S | 1.33 (d) | 22.7 | CH_3_ |
|  |  |  | 4.11 (q) | 71.3 | CH |
| **6** | Threonine | T and S | 1.32 | 22.1 | γ−CH_3_ |
|  |  |  | 3.57 | 63.3 | α−CH |
|  |  |  | 4.25 | 69.0 | β−CH |
| **7** | Alanine | T and S | 1.48 | 19.0 | CH_3_ |
|  |  |  | 3.78 | c | α CH |
| **8** | Lysine | T and S | 1.48 | 24.7 | γ−CH_2_ |
|  |  |  | 1.72 |  | δ−CH_2_ |
|  |  |  | 1.90 | 32.7 | β−CH_2_ |
|  |  |  | 3.03 | 42.1 | ε−CH_2_ |
|  |  |  | 3.73 | c | α−CH |
| **9** | Ornithine | S | 1.82,1.74 |  | γ-CH_2_ |
|  |  |  | 1.93 |  | β-CH_2_ |
|  |  |  | 3.08 (t) |  | δ-CH_2_ |
|  |  |  | 3.79 | c | α-CH |
| **10** | Arginine | S | 1.72,1.67 |  | γ-CH_2_ |
|  |  |  | 1.91 |  | β-CH_2_ |
|  |  |  | 3.25 |  | δ-CH_2_ |
|  |  |  | 3.76 | c | α-CH |
| **11** | Acetate | T and S | 1.92 (s) |  | CH_3_ |
| **12** | Acetone | S | 2.22 (s) | 32.8 | CH_3_ |
| **13** | N-acetyl | T and S | 2.06 (s) | 24.8 | CH_3_ |
| **14** | Methionine | T and S | 2.12 (s) | 17.1 | SCH_3_ |
|  |  |  | 2.65 (t) |  | γ−CH_2_ |
|  |  |  | 2.19 |  | β−CH_2_ |
|  |  |  | 3.84 |  | α−CH |
| **15** | Glutamate | T and S | 2.13, 2.06 | 29.2 | β−CH_2_ |
|  |  |  | 2.36 | 36.4 | γ−CH_2_ |
|  |  |  | 3.76 | c | α−CH |
| **16** | Glutamine | T and S | 2.13 | 29.0 | β−CH_2_ |
|  |  |  | 2.45 | 33.5 | γ−CH_2_ |
|  |  |  | 3.76 | c | α−CH |
| **17** | Proline | S | 2.01 |  | γ-CH_2_ |
|  |  |  | 2.35, 2.08 |  | β-CH_2_ |
|  |  |  | 3.43, 3.35 |  | δ-CH_2_ |
|  |  |  | 4.12 |  | α-CH |
| **18** | Pyroglutamic acid | T | 2.03, 2.52 | 27.9 | β-CH_2_ |
|  |  |  | 2.41 | 32.5 | γ-CH_2_ |
|  |  |  | 4.18 | 61.2 | α-CH |
| **19** | Pyruvate | S | 2.37 (s) |  | CH_3_ |
| **20** | Succinate | T | 2.41 (s) |  | CH_2_ |
| **21** | Glutathione | T | 2.16 |  | β-CH_2glu_ |
|  |  |  | 2.55 |  | γ-CH_2glu_ |
|  |  |  | 2.95 |  | β-CH_2cys_ |
|  |  |  | 4.57 |  | α-CH_cys_ |
| **22** | Citrate | S | 2.66 (d), 2.54 (d) | 48.5 |  |
| **23** | Aspartate | T | 2.68, 2.82 |  | β-CH_2_ |
|  |  |  | 3.90 |  | α-CH |
| **24** | Asparagine | S | 2.83, 2.94 |  | β-CH_2_ |
|  |  |  | 3.98 |  | α-CH |
| **25** | Dimethylamine | S | 2.74 (s) |  | CH_3_ |
| **26** | Dimethylglycine | S | 2.94 (s) | 45.4 | CH_3_ |
|  |  |  | 3.73 (s) | 62.9 | CH_2_ |
| **27** | Creatine | T and S | 3.04 (s) | 39.6 | CH_3_ |
|  |  |  | 3.93 (s) | 56.6 | CH_2_ |
| **28** | Creatinine | S | 3.05 (s) | 32.8 |  |
|  |  |  | 4.05 (s) | 59.8 | CH_2_ |
| **29** | S009 | S | 3.94 | 53.0 | CH_2_ |
|  |  |  | 3.64 | 51.8 | CH_2_ |
| **30** | Ethanolamine | T and S | 3.14 (t) |  | CH_2_ |
|  |  |  | 3.82 (t) |  | CH_2_ |
| **31** | Choline | T and S | 3.19 | 56.7 | N(CH_3_)_3_ |
|  |  |  | 3.50 |  | NCH_2_ |
|  |  |  | 4.06 |  | OCH_2_ |
| **32** | Phosphocholine | T | 3.22 | 56.7 | N(CH_3_)_3_ |
|  |  |  | 3.60 |  | NCH_2_ |
|  |  |  | 4.15 |  | OCH_2_ |
| **33** | Glycerophosphocholine | T and S | 3.23 (s) | 56.7 | N(CH_3_)_3_ |
| **34** | Taurine | T and S | 3.42 (t) | 38.2 | N-CH_2_ |
|  |  |  | 3.26 (t) | 50.2 | S-CH_2_ |
| **35** | Glycine | T and S | 3.56 (s) | 44.3 | CH_2_ |
| **35** | Glycerol | T | 3.56, 3.65 | 65.4 | 1-CH_2_ |
|  |  |  | 3.79 | 74.9 | 2-CH |
| **37** | Serine | T | 3.84 | 59.2 | α−CH |
|  |  |  | 3.97 | 63.2 | β−CH_2_ |
| **38** | Myo-inositol | T and S | 3.26 |  | 5-CH |
|  |  |  | 3.53 |  | 1,3-CH |
|  |  |  | 3.61 |  | 4,6-CH |
|  |  |  | 4.06 |  | 2-CH |
| **39** | Scyllo-inosito | T and S | 3.36 (s) |  | CH |
| **40** | Anhydrosorbitol | S | 3.35 |  | 4,5-CH |
|  |  |  | 3.45 |  | 3-CH |
|  |  |  | 3.61 |  | 2-CH |
|  |  |  | 3.70 |  | 6-CH |
|  |  |  | 3.28 (t), 3.99 |  | 1-CH_2_ |
| **41** | β-Glucose | T and S | 3.25(dd) | 77.0 | 2-CH |
|  |  |  | 3.41 (dd) | 72.4 | 4-CH |
|  |  |  | 3.47 | 78.6 | 5-CH |
|  |  |  | 3.50 (t) | 78.6 | 3-CH |
|  |  |  | 3.90 (dd), 3.73 | 63.6 | 6-CH_2_ |
|  |  |  | 4.65 (d) | 98.6 | 1-CH |
| **42** | α-Glucose | T and S | 3.42 (t) | 72.5 | 4-CH |
|  |  |  | 3.54 (dd) | 74.3 | 2-CH |
|  |  |  | 3.72 (t) | 75.6 | 3-CH |
|  |  |  | 3.83 | 74.2 | 5-CH |
|  |  |  | 3.85 (dd), 3.77 | 63.4 | 6 CH_2_ |
|  |  |  | 5.24 (d) | 94.8 | 1-CH |
| **43** | Ascorbate | T and S | 4.01 |  | 5-CH |
|  |  |  | 4.52 |  | 4-CH |
| **44** | α-Mannose | S | 3.94 |  | 2-CH |
|  |  |  | 5.19 (d) | 96.7 | 1-CH |
| **45** | β-Mannose | S | 4.90 (d) |  | 1-CH |
| **46** | Glycerol bound in TG | T | 4.31, 4.10 | 64.7 | CH_2_ |
|  |  |  | 5.22 | 71.7 | CH |
| **47** | Glycerol bound in PL | S | 3.22, 3.24 | 56.7 | N(CH_3_)_3_ |
|  |  |  | 3.66 | 68.7 | CH_2_ON(cho) |
|  |  |  | 4.30 | 62.2 | CH_2_OP(cho) |
|  |  |  | 3.88, 3.66 | 65.6 | CH_2_OP |
|  |  |  | 4.27, 4.06 | 64.4 | CH_2_OC=O |
|  |  |  | 5.12 | 71.6 | CHOC=O |
| **48** | Tyrosine | T and S | 3.19, 3.05 |  | β-CH_2_ |
|  |  |  | 3.94 |  | α-CH |
|  |  |  | 6.88 |  | 3-CH |
|  |  |  | 7.18 |  | 2-CH |
| **49** | Histidine | T and S | 3.19 |  | β-CH_2_ |
|  |  |  | 3.97 |  | α-CH |
|  |  |  | 7.05 (s) | 119.8 | 4-CH |
|  |  |  | 7.78 (s) |  | 2-CH |
| **50** | Phenylalanine | T and S | 3.28, 3.11 |  | β-CH_2_ |
|  |  |  | 3.99 |  | α-CH |
|  |  |  | 7.34 | 130.1 | H-*o* |
|  |  |  | 7.37 |  | H-*p* |
|  |  |  | 7.43 | 129.6 | H-*m* |
| **51** | Squalene^d^ | T | 1.58 | 18.8, 20.1 | CH_3_ |
|  |  |  | 1.66 | 28.4 | CH_3_ |
|  |  |  | 1.98, 2.03-2.07 |  | CH_2_CH=CH |
|  |  |  | 5.12-5.08 | 127.5 | HC=CH |
| **52** | Formate | S | 8.48 |  | CH |
| **53** | Ethanol^d^ | T | 1.18 |  | CH_3_ |
|  |  |  | 3.65 |  | CH_2_ |
| **54** | Isopropanol^d^ | T | 1.17 | 26.7 | CH_3_ |
|  |  |  | 4.03 |  | CH |
| **55** | Mepivacaine^d^ | T | 2.18 (bs) |  | CH_3_ |
|  |  |  | 2.23 (bs) |  | CH_3_ |
|  |  |  | 7.21 (d) |  | 3,5-CH |
|  |  |  | 7.25 (t) |  | 4-CH |

a,^1^H chemical shifts refer to Ala doublet at 1.48 ppm; b,^13^C chemical shifts refer to Ala at 19.0 ppm; c, contributes to the 3.77, 57.2 ppm cross-peak; d, metabolites or exogenous substances excluded from the study.
