# Supplementary material for: Integrated metabolomic analysis and cytokine profiling define clusters of immuno-metabolic correlation in new-onset psoriasis
Source: Sci Rep. 2021 May 18;11:10472. doi: 10.1038/s41598-021-89925-7 (PMC8131691; doi:10.1038/s41598-021-89925-7)
Supplement: Supplementary file 5 — Supplementary Information 5. [file 41598_2021_89925_MOESM5_ESM.docx]

|  | **PDGF-β** | **IL-1β** | **IL-1RA** | **IL-2** | **IL-4** | **IL-5** | **IL-6** | **IL-7** | **IL-8** | **IL-9** | **IL-13** | **IL-17** | **CCL11** | **bFGF** | **G-CSF** | **IFN-γ** | **CXCL10** | **CCL2** | **CCL3** | **CCL4** | **CCL5** | **TNF-α** | **VEGF** |
| --- | --- | --- | --- | --- | --- | --- | --- | --- | --- | --- | --- | --- | --- | --- | --- | --- | --- | --- | --- | --- | --- | --- | --- |
| **F** | -10 | 52 | 14 | 71 | 62 | 71 | -27 | 74 | 26 | 46 | 76 | 69 | 63 | 85 | 77 | 23 | 69 | -6 | 72 | 33 | 75 | 67 | 73 |
| **His** | 76 | 30 | -42 | -42 | -53 | -50 | -62 | -39 | -58 | -6 | -35 | -43 | -50 | -23 | -56 | -64 | -5 | -45 | -67 | 23 | -10 | -40 | -71 |
| **Phe** | -60 | -3 | 29 | -13 | -24 | -71 | 65 | -53 | 72 | -75 | -26 | -24 | -25 | -74 | -18 | 46 | -12 | 19 | 7 | -73 | -76 | -15 | -5 |
| **Man** | -65 | 52 | 23 | 6 | -10 | -37 | 22 | -16 | 28 | 4 | 13 | 2 | 6 | -19 | 34 | 54 | 38 | -5 | 44 | -0,9 | -30 | 7 | 30 |
| **Glc** | -55 | 13 | 30 | 89 | 68 | 72 | 33 | 73 | 39 | -3 | 83 | 72 | 62 | 60 | 73 | 49 | 42 | 21 | 91 | -26 | 53 | 80 | 93 |
| **Asc** | 44 | -9 | -52 | -65 | -73 | -71 | -7 | -79 | -14 | -50 | -45 | -74 | -74 | -66 | -78 | -54 | -48 | -50 | -70 | -24 | -62 | 67 | -77 |
| **Thr** | 32 | -41 | -29 | -66 | -51 | -57 | 22 | -84 | -1 | -64 | -59 | -63 | -54 | -65 | -67 | -33 | -57 | -21 | -63 | -42 | -72 | -76 | -65 |
| **Pro** | 32 | -62 | -50 | -71 | -33 | -0,5 | 12 | -57 | -26 | 4 | -38 | -57 | -35 | -24 | -44 | -43 | -70 | -33 | -52 | 10 | -40 | -79 | -46 |
| **Myo** | 5 | 86 | 5 | 5 | -18 | -49 | -43 | -9 | -25 | 8 | 6 | 1 | -10 | -5 | 9 | 6 | 58 | -26 | 7 | 23 | -0,7 | 11 | -7 |
| **Crn** | -58 | 23 | -19 | 14 | -3 | 12 | 21 | 11 | 26 | 31 | 53 | -4 | -0,9 | 6 | 30 | 21 | 6 | -36 | 49 | 15 | -1 | 14 | 41 |
| **Gly** | 20 | -44 | -29 | -91 | -63 | -71 | 24 | -99 | -0,5 | -42 | -77 | -80 | -62 | -80 | -71 | 26 | -66 | -20 | -72 | -23 | -89 | -95 | -75 |
| **ASP** | 69 | 12 | -37 | -75 | -55 | -47 | -66 | -45 | -70 | 44 | -62 | -56 | -47 | -25 | -52 | -57 | -18 | -32 | -80 | 65 | -16 | -64 | -80 |
| **GPC** | 39 | 41 | -25 | 54 | 31 | 64 | -56 | -66 | -49 | 40 | 67 | 43 | 29 | 78 | 34 | -31 | 42 | -33 | 30 | 39 | 82 | 50 | 33 |
| **Cho** | -32 | 68 | 13 | 51 | 40 | 41 | -29 | 59 | -18 | 67 | 65 | 49 | 45 | 63 | 72 | 31 | 69 | -13 | 68 | 53 | 56 | 55 | 63 |
| **Cr** | -29 | 75 | 42 | 61 | 33 | -8 | -8 | 33 | 12 | -16 | 39 | 51 | 35 | 23 | 50 | 46 | 78 | 14 | 57 | 16 | 24 | 60 | 47 |
| **Lys** | -71 | 23 | 88 | 67 | 82 | 35 | 40 | 49 | 38 | 0,2 | 27 | 81 | 83 | 40 | 89 | 96 | 69 | 72 | 85 | -21 | 23 | 66 | 86 |
| **Asn** | 23 | -29 | -52 | -91 | -76 | -70 | 10 | -95 | -9 | -26 | -59 | -89 | -74 | -76 | -74 | -42 | -67 | -48 | -71 | -7 | -81 | -94 | -77 |
| **DMG** | -73 | 23 | 39 | 62 | 58 | 39 | 46 | 37 | 41 | -7 | 61 | 55 | 56 | 35 | 74 | 69 | 46 | 18 | 89 | -27 | 15 | 53 | 85 |
| **DMA** | 23 | 11 | -22 | 65 | 29 | 70 | -30 | 76 | -13 | 17 | 71 | 43 | 23 | 65 | 21 | -29 | 18 | -17 | 30 | 9 | 79 | 63 | 37 |
| **Met** | -2 | -57 | 1 | -65 | -23 | -43 | 52 | -83 | 15 | -56 | -65 | -46 | -25 | -6 | -38 | 7 | -53 | 9 | -39 | -46 | -81 | -75 | -39 |
| **Pyr** | 18 | 38 | -15 | -42 | -58 | -88 | -16 | -63 | -7 | -38 | -40 | -49 | -54 | -61 | -48 | -17 | -0,3 | -29 | -43 | -14 | -54 | -40 | -56 |
| **Gln** | -9 | -66 | -25 | 5 | 4 | 32 | 56 | -10 | 37 | -56 | 20 | -10 | -8 | -8 | -17 | -12 | -55 | -10 | 6 | -61 | -17 | -11 | 11 |
| **NAc** | 8 | 73 | -13 | -11 | -47 | -62 | -50 | -9 | -15 | 21 | -5 | -25 | -39 | -26 | -20 | -16 | 31 | -31 | -18 | 34 | -4 | 4 | -31 |
| **Ac** | -33 | 19 | 29 | 93 | 74 | 84 | 9 | 88 | 17 | 14 | 83 | 81 | 69 | 78 | 75 | 37 | 51 | 22 | 84 | -7 | 74 | 87 | 89 |
| **Val** | -66 | 30 | 58 | -2 | 14 | 43 | 35 | -22 | 35 | -6 | 21 | 11 | 20 | -28 | 35 | 74 | 37 | 37 | 34 | -12 | -41 | 2 | 25 |
| **Ile** | -69 | 33 | 44 | 20 | 25 | -21 | 46 | -14 | 39 | -25 | 13 | 22 | 28 | -11 | 46 | 72 | 40 | 18 | 57 | -32 | -32 | 14 | 46 |

Table S2A. Correlation coefficients between metabolites and cytokines in serum samples of psoriatic patients.

|  | **PDGF-β** | **IL-1β** | **IL-1RA** | **IL-2** | **IL-4** | **IL-5** | **IL-6** | **IL-7** | **IL-8** | **IL-9** | **IL-13** | **IL-17** | **CCL11** | **bFGF** | **G-CSF** | **IFN-γ** | **CXCL10** | **CCL2** | **CCL3** | **CCL4** | **CCL5** | **TNF-α** | **VEGF** |
| --- | --- | --- | --- | --- | --- | --- | --- | --- | --- | --- | --- | --- | --- | --- | --- | --- | --- | --- | --- | --- | --- | --- | --- |
| **F** | -24 | -43 | 2 | -40 | -19 | -27 | -25 | -14 | 24 | -56 | -30 | -24 | -13 | -34 | 4 | -43 | -26 | -15 | 28 | -56 | -83 | -47 | -31 |
| **His** | 18 | -27 | -11 | -5 | -17 | -21 | -23 | -52 | -12 | 10 | -32 | -19 | -21 | -20 | -5 | -34 | -10 | -22 | 19 | 37 | 57 | -5 | -9 |
| **Phe** | -64 | 47 | 90 | 57 | 63 | 59 | 63 | 45 | 86 | 49 | 57 | 62 | 64 | 51 | 81 | 67 | 23 | 69 | 74 | 71 | 12 | 56 | 54 |
| **Man** | -6 | 90 | 12 | 85 | 87 | 91 | 88 | 82 | 63 | 90 | 86 | 89 | 86 | 92 | 73 | 52 | 72 | 80 | 47 | 71 | 40 | 90 | 95 |
| **Glc** | -60 | 80 | 75 | 88 | 83 | 81 | 83 | 70 | 90 | 75 | 82 | 83 | 82 | 82 | 74 | 91 | 65 | 87 | 56 | 83 | 43 | 82 | 78 |
| **Asc** | 27 | 22 | 23 | -13 | -7 | 8 | 13 | 26 | 28 | 10 | 23 | 4 | -6 | 0,2 | -2 | 46 | -52 | -1 | -47 | 2 | -14 | 13 | -3 |
| **Thr** | -43 | 66 | 77 | 70 | 68 | 69 | 72 | 54 | 89 | 71 | 69 | 71 | 67 | 65 | 74 | 80 | 32 | 72 | 54 | 87 | 42 | 74 | 66 |
| **Pro** | -42 | 91 | 51 | 82 | 93 | 95 | 95 | 98 | 82 | 73 | 95 | 94 | 94 | 92 | 86 | 77 | 63 | 93 | 56 | 58 | 1 | 85 | 89 |
| **Myo** | -41 | 67 | 78 | 50 | 57 | 63 | 69 | 74 | 84 | 47 | 74 | 63 | 59 | 57 | 60 | 94 | 10 | 66 | 20 | 47 | -6 | 60 | 51 |
| **Crn** | -33 | 67 | 73 | 47 | 55 | 63 | 68 | 75 | 82 | 47 | 74 | 62 | 57 | 56 | 58 | 92 | 6 | 63 | 15 | 44 | -9 | 59 | 50 |
| **Gly** | 24 | -38 | -66 | -15 | -36 | -43 | -49 | -57 | -65 | -20 | -50 | -42 | -41 | -29 | -60 | -56 | 29 | -44 | -27 | -20 | 47 | -33 | -30 |
| **ASo** | 17 | 4 | 22 | 3 | 7 | 12 | 13 | -8 | 29 | 30 | 6 | 11 | 7 | 2 | 32 | 2 | -33 | 5 | 27 | 48 | 25 | 21 | 15 |
| **GPC** | -26 | -36 | -24 | -7 | -24 | -37 | -40 | -43 | -45 | -35 | -41 | -34 | -27 | -24 | -45 | -30 | 39 | -25 | -8 | -27 | 19 | -37 | -31 |
| **Cho** | 92 | 8 | 85 | 22 | 29 | 18 | 23 | 25 | 48 | -14 | 23 | 23 | 31 | 15 | 38 | 52 | 18 | 42 | 46 | 4 | -39 | 3 | 6 |
| **Cr** | 51 | 49 | -42 | 40 | 22 | 32 | 27 | 22 | 4 | 61 | 32 | 27 | 16 | 41 | -9 | 20 | 39 | 12 | -34 | 44 | 81 | 50 | 40 |
| **Lys** | -7 | -43 | -17 | -36 | -51 | -55 | -54 | -42 | -48 | -55 | -47 | -54 | -52 | -45 | -69 | -13 | 7 | -45 | -55 | -53 | -6 | -53 | -58 |
| **Asn** | 26 | -77 | -12 | -72 | -76 | -76 | -74 | -82 | -47 | -58 | -76 | -75 | -76 | -80 | -54 | -51 | -76 | -73 | -34 | -33 | 7 | -67 | -75 |
| **DMG** | 33 | -11 | -36 | 9 | -15 | -16 | -21 | -46 | -23 | 23 | -25 | -16 | -21 | -7 | -27 | -27 | 17 | -24 | -9 | 38 | 87 | 5 | -2 |
| **DMA** | 46 | 19 | -9 | 15 | 3 | 12 | 11 | -10 | 16 | 49 | 9 | 10 | -0,7 | 11 | 5 | 8 | -12 | 3 | -9 | 59 | 73 | 34 | 20 |
| **Met** | 51 | -62 | -30 | -82 | -68 | -60 | -58 | -48 | -47 | -58 | -58 | -63 | -65 | -71 | -41 | -56 | -94 | -66 | -42 | -58 | -55 | -62 | -64 |
| **Pyr** | -68 | 6 | 87 | 5 | 15 | 12 | 19 | 24 | 51 | -16 | 21 | 15 | 19 | 3 | 33 | 57 | -19 | 30 | 25 | 2 | -47 | 0,5 | -3 |
| **Gln** | 66 | -48 | -38 | -78 | -75 | -62 | -59 | -43 | -50 | -51 | -51 | -67 | -74 | -67 | -67 | -32 | -89 | -72 | -86 | -58 | -31 | -55 | -67 |
| **NAc** | -25 | 63 | 71 | 42 | 50 | 58 | 63 | 68 | 80 | 48 | 69 | 57 | 51 | 50 | 55 | 88 | -2 | 57 | 11 | 47 | -3 | 58 | 47 |
| **Ac** | -30 | -11 | 17 | -23 | -0,03 | -3 | 0,7 | 64 | -1 | -42 | 2 | -2 | 6 | -10 | 12 | -2 | -18 | 7 | 11 | -55 | -93 | -26 | -14 |
| **Val** | 20 | 85 | 14 | 55 | 61 | 75 | 75 | 84 | 61 | 74 | 81 | 10 | 61 | 72 | 51 | 66 | 22 | 59 | 0,6 | 49 | 17 | 77 | 71 |
| **Ile** | 7 | 70 | 25 | 63 | 69 | 75 | 74 | 58 | 67 | 84 | 69 | 73 | 68 | 69 | 75 | 42 | 28 | 62 | 50 | 81 | 41 | 80 | -79 |

Table S2B. Correlation coefficients between metabolites and cytokines in serum samples of healthy subjects.

|  | **PDGF-β** | **IL-1β** | **IL-1RA** | **IL-2** | **IL-4** | **IL-5** | **IL-6** | **IL-8** | **IL-9** | **IL-10** | **IL-12 (p70)** | **IL-13** | **IL-15** | **IL-17** | **CCL11** | **bFGF** | **G-CSF** | **GM-CSF** | **IFN-γ** | **CXCL10** | **CCL2** | **CCL3** | **CCL4** | **CCL5** | **TNF-α** | **VEGF** |
| --- | --- | --- | --- | --- | --- | --- | --- | --- | --- | --- | --- | --- | --- | --- | --- | --- | --- | --- | --- | --- | --- | --- | --- | --- | --- | --- |
| **Glc** | 18 | -72 | -24 | 31 | 36 | -20 | -65 | -35 | -43 | 59 | 20 | 57 | 42 | -19 | -16 | -39 | -20 | -61 | -41 | -90 | -49 | -7 | -18 | -34 | 0,6 | -42 |
| **GSH** | 37 | 42 | 34 | 25 | 19 | 49 | 36 | 32 | 4 | 8 | 28 | -10 | -14 | 23 | 70 | -3 | 35 | 62 | 88 | 89 | 96 | 18 | 16 | -24 | 30 | 93 |
| **Asc** | -38 | -77 | 30 | 4 | -9 | -37 | -78 | -13 | -90 | -1 | -30 | -21 | -14 | -43 | -10 | -80 | -79 | -68 | 45 | -31 | 22 | -85 | -93 | -70 | -51 | 12 |
| **Thr** | -2 | -80 | -8 | 26 | 29 | -23 | -62 | -21 | -51 | 42 | 11 | 49 | 46 | -12 | -27 | -47 | -31 | -73 | -53 | -93 | -62 | -19 | -33 | -35 | -6 | -53 |
| **Ser** | -60 | -57 | -28 | -46 | -52 | -85 | -90 | -63 | -54 | -23 | -70 | -48 | -53 | -86 | -60 | -35 | -95 | -81 | 14 | -58 | -14 | -91 | -78 | -19 | -87 | -35 |
| **Gly** | -26 | -87 | 52 | 30 | 16 | -13 | -67 | 12 | -99 | 12 | -6 | 2 | 16 | -16 | 7 | -94 | -64 | -65 | 36 | -31 | -18 | -70 | -90 | -85 | -27 | 17 |
| **Myo** | -60 | -56 | -43 | -51 | -54 | -91 | -89 | -72 | -41 | -21 | -71 | -41 | -49 | -87 | -74 | -22 | -90 | -86 | -11 | -74 | -38 | -81 | -66 | -4 | -86 | -57 |
| **Scy** | -66 | 49 | -49 | -96 | -96 | -70 | 0,7 | -49 | 46 | -81 | -88 | -94 | -94 | -63 | -67 | 60 | -43 | -3 | 3 | 17 | -7 | -42 | -6 | 70 | -76 | -32 |
| **Tau** | -66 | -59 | -25 | -48 | -54 | -86 | -89 | -60 | -54 | -27 | -72 | -49 | -51 | -83 | -65 | -35 | -97 | -85 | 6 | -61 | -21 | -92 | -80 | -17 | -88 | -41 |
| **EA** | -45 | -89 | 0,4 | 4 | -9 | -53 | -79 | -26 | -68 | 3 | -30 | 5 | 13 | -38 | -52 | -58 | -73 | -97 | -38 | -90 | -58 | -65 | -73 | -37 | -47 | -59 |
| **Cr** | -60 | -68 | 17 | -22 | -35 | -58 | -79 | -25 | -77 | -25 | -55 | -44 | -35 | -58 | -35 | -64 | -92 | -76 | 33 | -35 | 8 | -96 | -95 | -48 | -72 | -8 |
| **Gln** | -8 | -65 | -34 | -4 | -6 | -57 | -97 | -68 | -60 | 32 | -25 | -0,9 | -24 | -74 | -23 | -45 | -64 | -64 | 27 | -62 | 5 | -59 | -55 | -43 | -52 | -9 |
| **PGA** | 12 | -95 | 22 | 52 | 48 | -5 | -74 | -6 | -86 | 58 | 26 | 51 | 48 | -9 | 10 | -84 | -37 | -66 | 1 | -68 | -11 | -33 | -56 | -80 | 0,4 | -4 |
| **Glu** | 13 | -92 | 54 | 67 | 58 | 19 | -55 | 26 | -94 | 51 | 37 | 51 | 59 | 15 | 31 | -96 | -28 | -52 | 15 | -42 | 7 | -31 | -62 | -93 | 15 | 18 |
| **Met** | -83 | -50 | 47 | -30 | -46 | -41 | -36 | 17 | -61 | -61 | -58 | -57 | -23 | -22 | -41 | -53 | -81 | -66 | 8 | -14 | -11 | -88 | -91 | -29 | -61 | -20 |
| **NAc** | -47 | -83 | 0,9 | -6 | -9 | -49 | -66 | -19 | -57 | -2 | -28 | 8 | 20 | -27 | -56 | -49 | -65 | -94 | -55 | -91 | -72 | -55 | -63 | -25 | -41 | -70 |
| **Ac** | 8 | -62 | -48 | 8 | 14 | -44 | -77 | -62 | -34 | 49 | 2 | 37 | 13 | -47 | -32 | -24 | -34 | -63 | -33 | -92 | -45 | -19 | -19 | -20 | -22 | -47 |
| **Lys** | 77 | -46 | 28 | 93 | 95 | 61 | -12 | 27 | -40 | 93 | 87 | 97 | 85 | 47 | 66 | -52 | 44 | 6 | 3 | -23 | 12 | 45 | 14 | -67 | 72 | 34 |
| **Ala** | 24 | -56 | 48 | 55 | 43 | 21 | -48 | 14 | -81 | 44 | 29 | 22 | 18 | -3 | 54 | -82 | -23 | -14 | 77 | 8 | 68 | -34 | -54 | -91 | -7 | 67 |
| **Val** | -35 | -90 | 7 | -2 | -6 | -55 | -86 | -35 | -70 | 11 | -28 | 7 | 9 | -45 | -48 | -58 | -73 | -96 | -30 | -91 | -50 | -64 | -71 | -40 | -48 | -54 |

Table S2C. Correlation coefficients between metabolites and cytokines in tissue samples of psoriatic patients.

|  | **PDGF-α** | **IL-1β** | **IL-1RA** | **IL-2** | **IL-4** | **IL-5** | **IL-6** | **IL-8** | **IL-9** | **IL-10** | **IL-12 (p70)** | **IL-13** | **IL-15** | **IL-17** | **CCL11** | **bFGF** | **G-CSF** | **GM-CSF** | **IFN-γ** | **CXCL10** | **CCL2** | **CCL3** | **CCL4** | **CCL5** | **TNF-α** | **VEGF** |
| --- | --- | --- | --- | --- | --- | --- | --- | --- | --- | --- | --- | --- | --- | --- | --- | --- | --- | --- | --- | --- | --- | --- | --- | --- | --- | --- |
| **Glc** | 18 | -72 | -24 | 31 | 36 | -20 | -65 | -35 | -43 | 59 | 20 | 57 | 42 | -19 | -16 | -39 | -20 | -61 | -41 | -90 | -49 | -7 | -18 | -34 | 0,6 | -42 |
| **GSH** | 37 | 42 | 34 | 25 | 19 | 49 | 36 | 32 | 4 | 8 | 28 | -10 | -14 | 23 | 70 | -3 | 35 | 62 | 88 | 89 | 96 | 18 | 16 | -24 | 30 | 93 |
| **Asc** | -38 | -77 | 30 | 4 | -9 | -37 | -78 | -13 | -90 | -1 | -30 | -21 | -14 | -43 | -10 | -80 | -79 | -68 | 45 | -31 | 22 | -85 | -93 | -70 | -51 | 12 |
| **Thr** | -2 | -80 | -8 | 26 | 29 | -23 | -62 | -21 | -51 | 42 | 11 | 49 | 46 | -12 | -27 | -47 | -31 | -73 | -53 | -93 | -62 | -19 | -33 | -35 | -6 | -53 |
| **Ser** | -60 | -57 | -28 | -46 | -52 | -85 | -90 | -63 | -54 | -23 | -70 | -48 | -53 | -86 | -60 | -35 | -95 | -81 | 14 | -58 | -14 | -91 | -78 | -19 | -87 | -35 |
| **Gly** | -26 | -87 | 52 | 30 | 16 | -13 | -67 | 12 | -99 | 12 | -6 | 2 | 16 | -16 | 7 | -94 | -64 | -65 | 36 | -31 | -18 | -70 | -90 | -85 | -27 | 17 |
| **Myo** | -60 | -56 | -43 | -51 | -54 | -91 | -89 | -72 | -41 | -21 | -71 | -41 | -49 | -87 | -74 | -22 | -90 | -86 | -11 | -74 | -38 | -81 | -66 | -4 | -86 | -57 |
| **Scy** | -66 | 49 | -49 | -96 | -96 | -70 | 0,7 | -49 | 46 | -81 | -88 | -94 | -94 | -63 | -67 | 60 | -43 | -3 | 3 | 17 | -7 | -42 | -6 | 70 | -76 | -32 |
| **Tau** | -66 | -59 | -25 | -48 | -54 | -86 | -89 | -60 | -54 | -27 | -72 | -49 | -51 | -83 | -65 | -35 | -97 | -85 | 6 | -61 | -21 | -92 | -80 | -17 | -88 | -41 |
| **EA** | -45 | -89 | 0,4 | 4 | -9 | -53 | -79 | -26 | -68 | 3 | -30 | 5 | 13 | -38 | -52 | -58 | -73 | -97 | -38 | -90 | -58 | -65 | -73 | -37 | -47 | -59 |
| **Cr** | -60 | -68 | 17 | -22 | -35 | -58 | -79 | -25 | -77 | -25 | -55 | -44 | -35 | -58 | -35 | -64 | -92 | -76 | 33 | -35 | 8 | -96 | -95 | -48 | -72 | -8 |
| **Gln** | -8 | -65 | -34 | -4 | -6 | -57 | -97 | -68 | -60 | 32 | -25 | -0,9 | -24 | -74 | -23 | -45 | -64 | -64 | 27 | -62 | 5 | -59 | -55 | -43 | -52 | -9 |
| **PGA** | 12 | -95 | 22 | 52 | 48 | -5 | -74 | -6 | -86 | 58 | 26 | 51 | 48 | -9 | 10 | -84 | -37 | -66 | 1 | -68 | -11 | -33 | -56 | -80 | 0,4 | -4 |
| **Glu** | 13 | -92 | 54 | 67 | 58 | 19 | -55 | 26 | -94 | 51 | 37 | 51 | 59 | 15 | 31 | -96 | -28 | -52 | 15 | -42 | 7 | -31 | -62 | -93 | 15 | 18 |
| **Met** | -83 | -50 | 47 | -30 | -46 | -41 | -36 | 17 | -61 | -61 | -58 | -57 | -23 | -22 | -41 | -53 | -81 | -66 | 8 | -14 | -11 | -88 | -91 | -29 | -61 | -20 |
| **NAc** | -47 | -83 | 0,9 | -6 | -9 | -49 | -66 | -19 | -57 | -2 | -28 | 8 | 20 | -27 | -56 | -49 | -65 | -94 | -55 | -91 | -72 | -55 | -63 | -25 | -41 | -70 |
| **Ac** | 8 | -62 | -48 | 8 | 14 | -44 | -77 | -62 | -34 | 49 | 2 | 37 | 13 | -47 | -32 | -24 | -34 | -63 | -33 | -92 | -45 | -19 | -19 | -20 | -22 | -47 |
| **Lys** | 77 | -46 | 28 | 93 | 95 | 61 | -12 | 27 | -40 | 93 | 87 | 97 | 85 | 47 | 66 | -52 | 44 | 6 | 3 | -23 | 12 | 45 | 14 | -67 | 72 | 34 |
| **Ala** | 24 | -56 | 48 | 55 | 43 | 21 | -48 | 14 | -81 | 44 | 29 | 22 | 18 | -3 | 54 | -82 | -23 | -14 | 77 | 8 | 68 | -34 | -54 | -91 | -7 | 67 |
| **Val** | -35 | -90 | 7 | -2 | -6 | -55 | -86 | -35 | -70 | 11 | -28 | 7 | 9 | -45 | -48 | -58 | -73 | -96 | -30 | -91 | -50 | -64 | -71 | -40 | -48 | -54 |

Table S2D. Correlation coefficients between metabolites and cytokines in tissue samples of healthy subjects.
